# Human monocyte subtype expression of neuroinflammation- and regeneration-related genes is linked to age and sex

**DOI:** 10.1371/journal.pone.0300946

**Published:** 2024-10-30

**Authors:** Juliane F. Tampé, Emanuela Monni, Sara Palma-Tortosa, Emil Brogårdh, Charlotta Böiers, Arne G. Lindgren, Zaal Kokaia

**Affiliations:** 1 Laboratory of Stem Cells and Restorative Neurology, Lund Stem Cell Center, Lund University, Lund, Sweden; 2 Department of Neurology, Skåne University Hospital; Department of Clinical Sciences Lund, Neurology, Lund University, Lund, Sweden; 3 Division of Molecular Hematology, Lund Stem Cell Center, Lund University, Lund, Sweden; University of Virginia, UNITED STATES OF AMERICA

## Abstract

Aging profoundly affects the immune system leading to an increased propensity for inflammation. Age-related dysregulation of immune cells is implicated in the development and progression of numerous age-related diseases such as: cardiovascular diseases, neurodegenerative disorders, and metabolic syndromes. Monocytes and monocyte-derived macrophages, being important players in the inflammatory response, significantly influence the aging process and the associated increase in inflammatory disease risk. Ischemic stroke is among age-related diseases where inflammation, particularly monocyte-derived macrophages, plays an important deteriorating role but could also strongly promote post-stroke recovery. Also, biological sex influences the incidence, presentation, and outcomes of ischemic stroke, reflecting both biological differences between men and women. Here, we studied whether human peripheral blood monocyte subtype (classical, intermediate, and non-classical) expression of genes implicated in stroke-related inflammation and post-stroke tissue regeneration depends on age and sex. A flow cytometry analysis of blood samples from 44 healthy volunteers (male and female, aged 28 to 98) showed that in contrast to other immune cells, the proportion of NK-cells increased in females. The proportion of B-cells decreased in both sexes with age. Gene expression analysis by qPCR identified several genes differentially correlating with age and sex within different monocyte subtypes. Interestingly, *ANXA1* and *CD36* showed a consistent increase with aging in all monocytes, specifically in intermediate (*CD36*) and intermediate and non-classical (*ANXA1*) subtypes. Other genes (*IL-1β*, *S100A8*, *TNFα*, *CD64*, *CD33*, *TGFβ1*, *TLR8*, *CD91*) were differentially changed in monocyte subtypes with increasing age. Most age-dependent gene changes were differentially expressed in female monocytes. Our data shed light on the nuanced interplay of age and sex in shaping the expression of inflammation- and regeneration-related genes within distinct monocyte subtypes. Understanding these dynamics could pave the way for targeted interventions and personalized approaches in post-stroke care, particularly for the aging population and individuals of different sexes.

## Introduction

Aging is associated with alterations in the immune system, which can lead to an increased susceptibility to various diseases and diminished immune responses. This is largely due to the cumulative effects of age-related changes in the immune system involving a gradual decline in immune function. This makes the body more susceptible to chronic low-grade inflammation, known as "inflammaging", a well-studied, chronic, sterile inflammatory phenotype in aging research [1]. Additionally, the accumulation of cellular damage and the presence of comorbid conditions, such as hypertension, diabetes, and atherosclerosis further exacerbate inflammatory responses. These factors collectively contribute to the increased incidence of inflammatory diseases in the elderly. Monocytes and monocyte-derived macrophages (MDMs) together with other immune cells, play a crucial role in inflammaging. These cells have also been linked to functional immune decline associated with age-related diseases such as atherosclerosis, diabetes, fibrosis, immunodeficiency, autoimmunity, and cancer [2]. Recently, Moss and co-workers reported an age-related decrease in migration and phagocytosis in human monocytes and MDMs. They found that this characteristic of the aged macrophage phenotype is influenced by the master-regulator transcription factors MYC and USF1 in both human and murine cells [3].

As individuals age, together with other diseases, the risk of stroke rises, making it predominantly an age-related disease. Moreover, it is well-established that there are sex-related differences in both occurrence and recovery after stroke. Inflammation plays a crucial role in the pathophysiology of ischemic stroke by promoting atherosclerotic plaque formation, destabilization, and subsequent thrombosis, which can obstruct cerebral blood flow and result in stroke. Our recent research suggests that peripheral blood monocytes at different time points after a stroke could contribute to regenerative potential [4]. Monocytes are a heterogeneous cell population [5], with pro- or anti-inflammatory phenotypes depending on the stage of differentiation and mechanism by which they are activated [6]. Upon brain damage such as traumatic injury or stroke, immune cells from the blood, including monocytes, may enter the brain tissue [7] and contribute to neuroinflammation and regeneration of brain function [8–10]. We previously showed [4] that depletion of MDMs during the first week after stroke in an animal model abolished long-term behavioral recovery and drastically decreased tissue expression of anti-inflammatory genes, including transforming growth factor (*TGFβ1*), cluster of differentiation 163 (*CD163*), and chitinase-3-like protein 3 (*Ym1*). Our observations suggest that MDMs play an essential role in post-stroke recovery by activating anti-inflammatory factors. In support, we later reported that mouse MDMs, primed *in vitro* to become anti-inflammatory macrophages and then administered into the cerebrospinal fluid of stroke-subjected mice, infiltrate the ischemic hemisphere and promote post-stroke recovery of motor and cognitive functions [11].

Interestingly, different subtypes of human monocytes have been correlated with outcome severity and prognosis in patients following stroke [12]. Human monocytes are categorized into three subtypes based on the expression of the specific cell surface markers CD14 and CD16 and functional characteristics [13]. Classical monocytes (CD14+/CD16-) [14] represent about 85% of the total number [15] and possess phagocytic and antigen-presenting capabilities. Non-classical monocytes (CD14-/CD16+) constitute about 10% and have patrolling functions and a role in the early immune response to infections [16]. Intermediate monocytes (CD14+/CD16+) comprise about 5% of the total number of monocytes [17, 18] and are potent producers of inflammatory cytokines, contributing to acute and chronic inflammation [19]. Considering the involvement of monocytes in stroke outcomes, it seems highly warranted to explore how age and sex influence the expression of inflammation-related genes in the different monocyte subtypes, which is currently poorly understood [20, 21].

Monocytes, like most cells in the human body, do not have a defined age. However, monocytes have a limited life span once differentiated from hematopoietic progenitor cells in the bone marrow [16]. It is still unclear whether the phenotypic and genetic profiles of peripheral monocytes, like most cells in the human body, are related to the monocyte age. In addition, their abundance and function may be affected by the age and sex of the patient. The number of monocytes in circulation may decrease with age [22], and the function of monocytes may also decline, impairing the immune response and increasing susceptibility to infections [23]. Sex may also play a role in the count and function of monocytes [24]. Studies have found that women generally have higher numbers of circulating monocytes than men, but the function of monocytes may be more robust in men than in women [25]. Monocyte counts may also be related to ethnicity [26]. Overall, the effects of age and sex on monocytes are complex and can be influenced by various factors, such as genetics, lifestyle, and environmental exposures [27]. It is unclear whether the age and sex of patients can influence the expression of specific genes linked to the inflammation-caused exaggeration of ischemic damage and promotion of regeneration and functional recovery [20, 21]. Further research is needed to fully understand the properties of monocytes from age and sex perspectives [28].

Here, we have determined how aging affects the expression of neuroinflammation-related genes in subtypes of human monocytes in a group of 26 male and 18 female healthy volunteers aged 28–98 years. We find several genes that are up- or downregulated with age. When the findings in male and female subjects were analyzed separately, we observed that most age-related gene changes occur in females.

## Materials and methods

### Ethical approval for human subjects

All procedures were carried out in accordance with the ethical standards of the Regional Ethics Testing Agency in Lund, Sweden. The study protocol was approved under the Lund Stroke Register project (ethical permit diary number 2016/179; approved on 4.8.2016 and 2017/357; approved on 2.5.2017) in compliance with the Declaration of Helsinki. The ethical permit covers, among other procedures, a blood sampling volume of a maximum of 80 mL across two occasions per healthy donor. Informed, written consent for blood sampling, genetic analysis, protein analysis, and clinical assessments was obtained from all participants included in the study. Participants received detailed information about the study, all procedures involved, and the possibility of discontinuing the study at any point to ensure transparency and full consent. In case of exclusion from the study, already collected samples were destroyed and discarded. Participants were required to be healthy at the time of blood donation. Pregnancy, systemic inflammatory diseases (e.g., rheumatoid arthritis, systemic lupus erythematosus), or hematologic diseases affecting the immunological status served as exclusion criteria.

This cohort study includes no intervention except venous blood sampling, carried out using standardized, routine methods. All samples were coded not to carry any personal identifier information. To avoid potential bias, samples were randomized. Data collection and analysis were performed blindly.

### Collection of peripheral blood mononuclear cells

Specialized research nurses at the Department of Neurology at Skåne University Hospital collected 10mL of venous blood in heparin-treated, ethylenediaminetetraacetic acid (EDTA) coated vacutainers (Sarstedt, Germany). The blood samples were collected from male and female volunteers aged between 28 and 98 years (S2 Table 1 in S2 File). After collection, the blood samples were kept at room temperature (RT) before mononuclear cell isolation within 24h. Peripheral blood mononuclear cells (PBMCs) were isolated in a biosafety level 2+ cell laboratory under constant airflow in a laminar flow hood. All reagents used were devoid of animal-derived products. Human recombinant albumin (HSA, Merck, Sweden) was used in place of fetal bovine serum to avoid any trigger of monocyte activation. All procedures were performed at RT. According to the manufacturer’s instructions, PBMCs were isolated using SepMate tubes (StemCell Technologies, Sweden) containing Lymphoprep (Serumwerk, Germany) by density gradient centrifugation. PBMCs were frozen in StemCellBanker (Amsbio, UK) at -80 °C. The next day, samples were cryopreserved in liquid nitrogen (-170°C) or a -150°C freezer for long-term storage until further analysis or Fluorescence-Activated Cell Sorting (FACS).

### Flow cytometry and FACS preparation

The frozen PBMCs were thawed at RT and washed twice in 10 mL buffer. Dulbecco’s Phosphate-Buffered Saline (DPBS) with 2% HSA was used as a buffer during all procedures. The pelleted cells were then resuspended in 60 μL buffer and incubated with the appropriate antibody concentration on a shaker for 30–60 min at 4°C. Then, cells were washed twice with 1 mL of buffer and centrifuged at 500 x g for 5 min. Finally, the resulting pellet was resuspended in 200 μL buffer and strained through the 35 μm mesh incorporated in a tube for flow cytometry applications (Falcon, Corning, USA).

Primary human monocytes and their subtypes were identified and isolated from PBMCs based on their surface expression of CD91 [29] and their differential expression of the CD14 and CD16 cell surface markers. The B-, natural killer (NK)- and T-cells were identified based on their CD19, CD56, and CD3 expression. The following antibodies were employed: CD91-PE (clone A2MR-α2), CD14-APC (clone M5E2), CD16-BV421 (clone 3G8), CD3-PE-Cy7 (clone UCHT1), CD19-BB515 (clone HIB19), CD56-BV605 (clone B159), (BD Biosciences, Sweden). To exclude non-viable cells, DRAQ7 (BD Biosciences, Sweden) was added to the cell suspension 15 min before analysis (S2 Table 2 in S2 File). Cell type identification and isolation were performed using the FACS system Aria^™^ II (BD Biosciences, Sweden).

We first established a baseline of the autofluorescence using cells that were not subjected to any staining procedures as a negative control. Single-stained samples were used to compensate for the fluorophores’ spectral overlap. We utilized fluorescence minus one (FMO) to define the gating strategy. All cell population types have additionally been confirmed by FMOs, re-analysis, and back gating. The antibodies and staining volumes have been scaled according to the number of PBMCs to achieve the same staining concentration in all samples (S2 Table 3 in S2 File).

### FACS analysis and sorting

The cells were separated from debris and selected for size using the area of the forward and side scatter (FSC and SCC, respectively). We excluded doublets by using FSC-W/H and SCC-W/H, and eliminated dead cells by utilizing the intracellular dye DRAQ7. The three monocyte subpopulations were further classified by the expression of CD14 and CD16: CD14+ and CD16- classical monocytes, CD14-and CD16+ non-classical monocytes, and CD14+ and CD16+ intermediate monocytes. For monocytes and each of the three subtypes, biological duplicates of 20 cells from the same donor were sorted into each well of a 96-well-cell culture plate (Corning, Sweden). Before cell sorting, a lysis buffer consisting of 10% NP40 (Thermo Fisher, Sweden), 10mM dNTP (Takara, Japan), 0.1M DTT (Thermo Fisher Scientific, Sweden), RNaseOUT (Thermo Fisher Scientific, Sweden), and nuclease-free water was dispensed to a 96-well-cell culture plate. The plate was spun at 1300 rpm and kept at -80°C for subsequent pre-amplification and analysis using quantitative polymerase chain reaction (qPCR).

### High-throughput microfluidics technology quantitative PCR (Fluidigm)

To analyze the potential age- and sex-related differences in gene expression between the monocytes and their subtypes, we performed a Fluidigm-based study and examined the expression of 40 brain inflammation- and regeneration-related genes. Fluidigm is a microfluidics technology that allows simultaneous and efficient gene expression analysis using integrated fluidic circuits (IFCs) [30]. The IFC performs precise and high-throughput quantification of mRNA levels of up to 96 genes. The small format reduced sample and reagent requirements, allowing sensitive measurements of low mRNA samples. The chosen regenerative genes were selected through an extensive literature review, focusing on genes expressed by monocytes and/or macrophages and implicated in neuroinflammation and the context of stroke recovery. Three housekeeping genes, four negative controls for the other PBMC populations, and one technical control were deployed to normalize the expression and do quality control (S2 Table 6 in S2 File).

The complementary DNA (cDNA) was generated with each of the 47 TaqMan probes (S1 Table 6 in S2 File) and Xeno primer (TaqMan Cells-to-Ct Control kit, Thermo Fisher Scientific, Sweden) and using the Taq-SSIII reaction mix (CellsDirectTM One-Step qRT-PCR Kit, Ambion, Thermo Fisher Scientific, Sweden). The negative, positive, no reverse transcriptase (noRT), and linearity controls were included on two plates and only repeated when a new batch of probes or SSIII enzyme was used. We used a real-time PCR (rtPCR) starting with an extended 50°C for 1 h followed by 2 min at 95°C. For the panel of primers used, we identified that 18 cycles at 95°C for 15 seconds and 60°C for 4 min were optimal for pre-amplification. The produced cDNA was used directly for rtPCR or stored at -80°C.

The TaqMan Gene Expression Master Mix (Thermo Fisher Scientific, Sweden) and GE Sample loading reagent (100–7610, Fluidigm, Standard Biotools Inc., California) were mixed 10:1, and 3.3 μL of the mix was added to each well of a 96-well plate. Next, the cDNA from the pre-amplification was diluted 1:5 and 2.7 μL added to the pre-mixed sample plate for a final volume of 6 μL. Once all sample and assay mixes were prepared, the Integrated Fluidic Circuit (IFC) chip was injected with the control line fluid via the two valves on the chip. The IFC chip was then inserted into the Integrated Fluidic Unit (IFU) and primed using pressure to push the control line fluid into all reaction chambers and channels, ensuring they were free from air bubbles and debris. Once primed, the chip was loaded by pipetting at least 4 μL of either sample or assay mix into the corresponding wells of the chip. Qualitative gene expression was measured by detecting FAM-MGB during a cycle of 50-70-25-50-96. We set a baseline for the lowest and threshold fluorescence signals using the BioMark HD Data Analysis software (Standard Biotools Inc., California). The resulting cycle threshold (CT) values were normalized against the beta-actin (*ACTβ*) reference gene. The Fluidigm data were used to conduct the linear regression analysis and identify potential correlations between age and the expression of selected genes linked to the monocytes’ function or activation mode and their subtypes or their inflammatory response during brain injury.

### Statistical analysis

In this exploratory study, PBMC samples were block-randomized and blinded before any analysis, so no biases are to be declared. One million events per sample were collected using the FACSAria^™^II cell sorter, operated by the DIVA software. The data were analyzed using the software FlowJo (Version 10.10.0) to estimate the overall cell population and visualize the gating strategy (Fig 2). The group comparison between the Adult and Older group (above 65 years), or male and female, was assessed by an unpaired t-test using the statistical software PRISM 10 (GraphPad).

The rtPCR data of each Fluidigm IFC was analyzed using the BioMark HD analysis software with a quality threshold of 0.65, linear baseline correction, and automatic global cycle threshold. The data from all chips, metadata, and flow cytometry data were pooled using the software R (https://github.com/JTampe/Monocytes). The age distribution histogram and cell type percentages of all donors were normally distributed (data not shown). Non-detected runs were set as CT value = 35 [31], and failed runs were multiplied and imputed using Gene, Subtype, Sex, and Age [32]. We normalized both technical and biological duplicates using *ACTβ* expression and calculated the mean relative expression (rE) from these four values. The association of age with gene expression was assessed using Pearson correlation. The independent, normally distributed observations were checked for non-linear patterns and variance. The residual analysis-identified outliers, defined as values more than 3 times the standard deviation, were excluded from the final analysis to ensure robust statistical conclusions. Finally, a linear regression model was fitted (relative expression ~ age) for each gene and subpopulation. The Pearson correlation coefficient (r, displayed in Fig 6) and its confidence interval were extracted from the regression model. We also extracted the slope, its 95% confidence interval, and intercept from the model (reported in S2 Table 8 in S2 File). A p-value < 0.05 was considered statistically significant. For significant age associations, a fitted linear regression was plotted with a 95% confidence interval, describing the true mean of the linear correlation with 95% certainty.

### Graphics and design

**Fig 1:**
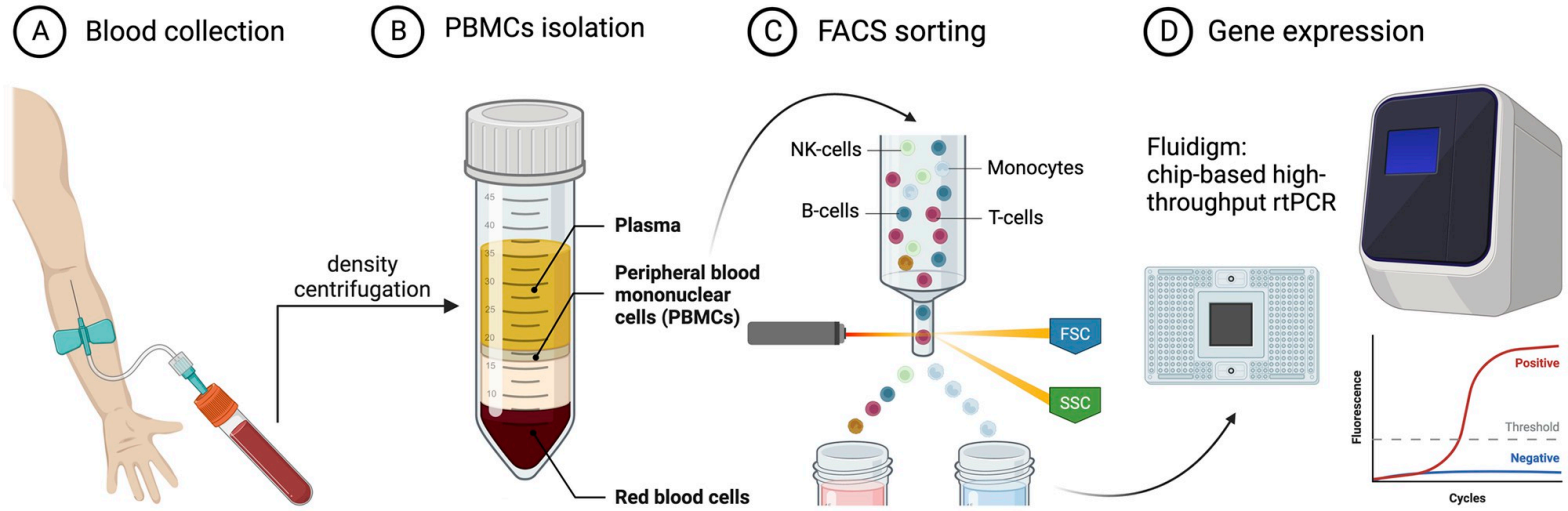
Workflow of the study. **(A**) Blood sample collection. (**B**) Isolation of peripheral blood mononuclear cells. (**C**) Flow cytometry analysis and isolation of monocytes. (**D**) Gene expression analysis using Fluidigm—a multiplex qPCR. This image was generated using BioRender.com.

**Fig 2:**
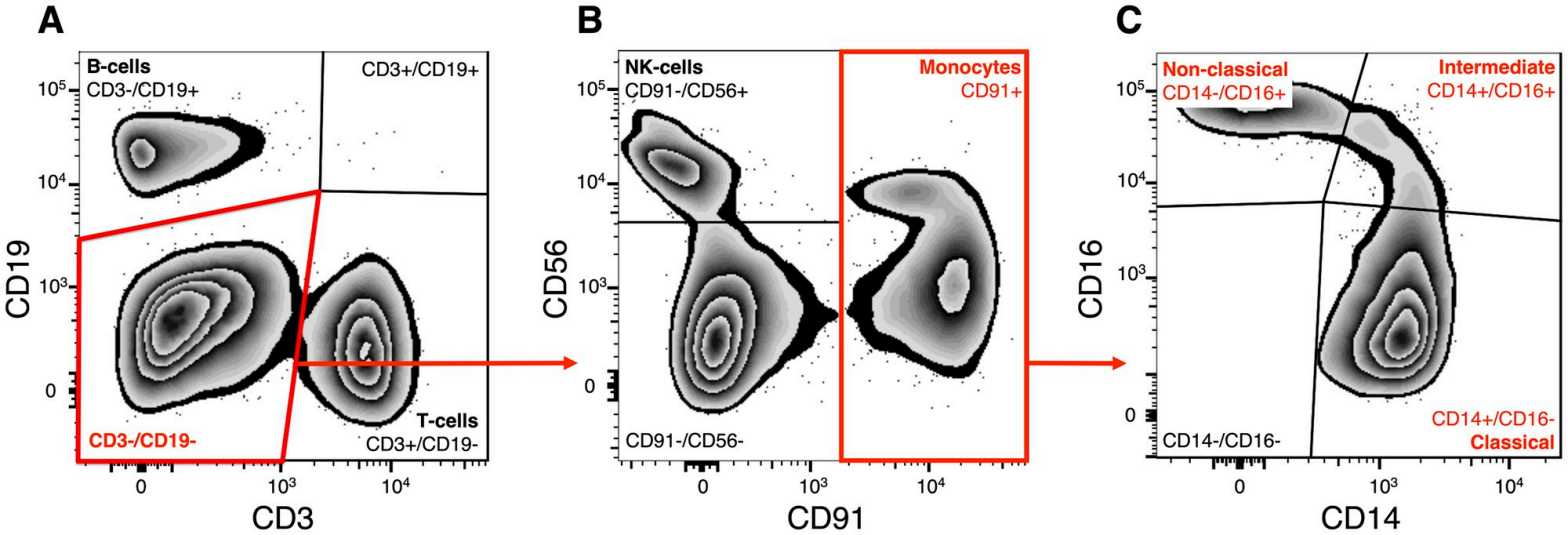
FACS gating strategy for isolated PBMCs, followed by sorting and analysis of the three monocyte subtypes. Single cells negative for Draq7 were considered viable. Cells negative for (**A**) T-cell marker CD3 and B-cell marker CD19 were excluded. (**B**) NK-cells were defined as CD56+CD91-. The CD91 positive cells were gated further and (**C**) sub-fractioned as classical (CD14+/CD16-), intermediate (CD14+/CD16+), and non-classical (CD14-/CD16+) monocytes.

BioRender.com was used to create Fig 1, FlowJo (Version 10.10.0) for the visualization of the gating strategy in Fig 2, and PRISM (Version 10, GraphPad) was used to plot PBMC populations in S1 Fig 1 in S1 File. The heatmap in Fig 6, showing the Pearson correlation coefficient, was generated using the matrix visualization and analysis software Morpheus (https://software.broadinstitute.org/morpheus). All other figures and tables were generated using the software R (R Core Team (2021). R: A language and environment for statistical##computing. R Foundation for Statistical Computing, Vienna, Austria. ##URL https://www.R-project.org/.).

## Results

In this study, we aimed to investigate age-dependent changes in immune cell counts and gene expression levels. The study’s main flow and different steps are presented in Fig 1. To ensure balanced and representative samples, we randomly selected a relatively equal number of male and female blood samples from our available pool of donors, achieving statistically comparable average ages across sexes. Additionally, participants were chosen to represent a broad age range, with a normal distribution across this spectrum, allowing us to effectively examine the influence of age on the expression of genes in peripheral monocytes. The blood samples were obtained from a total of 44 volunteers (18 females (41%) and 26 males (59%) (S2 Table 1 in S2 File). The median age for the females was 72 years (range: 43 to 98), while the median age for the males was 66 (range of 28 to 87 years). The average ages were not significantly different between male (n = 26) and female (n = 18) groups (Unpaired t-test; p = 0.0634).

### The proportions of B- and NK-cells are linked to age and sex

After isolating PBMCs, we performed a flow cytometry analysis to compare the relative percentages of different PBMCs expressed as a proportion of all PBMCs in the age- and sex-dependent groups. First, we analyzed the expression of *CD19* and *CD3* in PBMCs (Fig 2A), which allowed us to quantify the proportions of B- (CD3-/CD19+) and T- (CD3+/CD19-) cells. Then, we flow analyzed CD3-/CD19- cells for the expression of *CD56* and *CD91* (Fig 2B) and quantified the proportion of NK-cells (CD56+/CD91-) and monocytes (CD91+). Finally, to gain a deeper insight into the functional heterogeneity of the monocyte population, we analyzed subtypes of monocytes based on *CD16* and *CD14* expression (Fig 2C).

**Fig 3:**
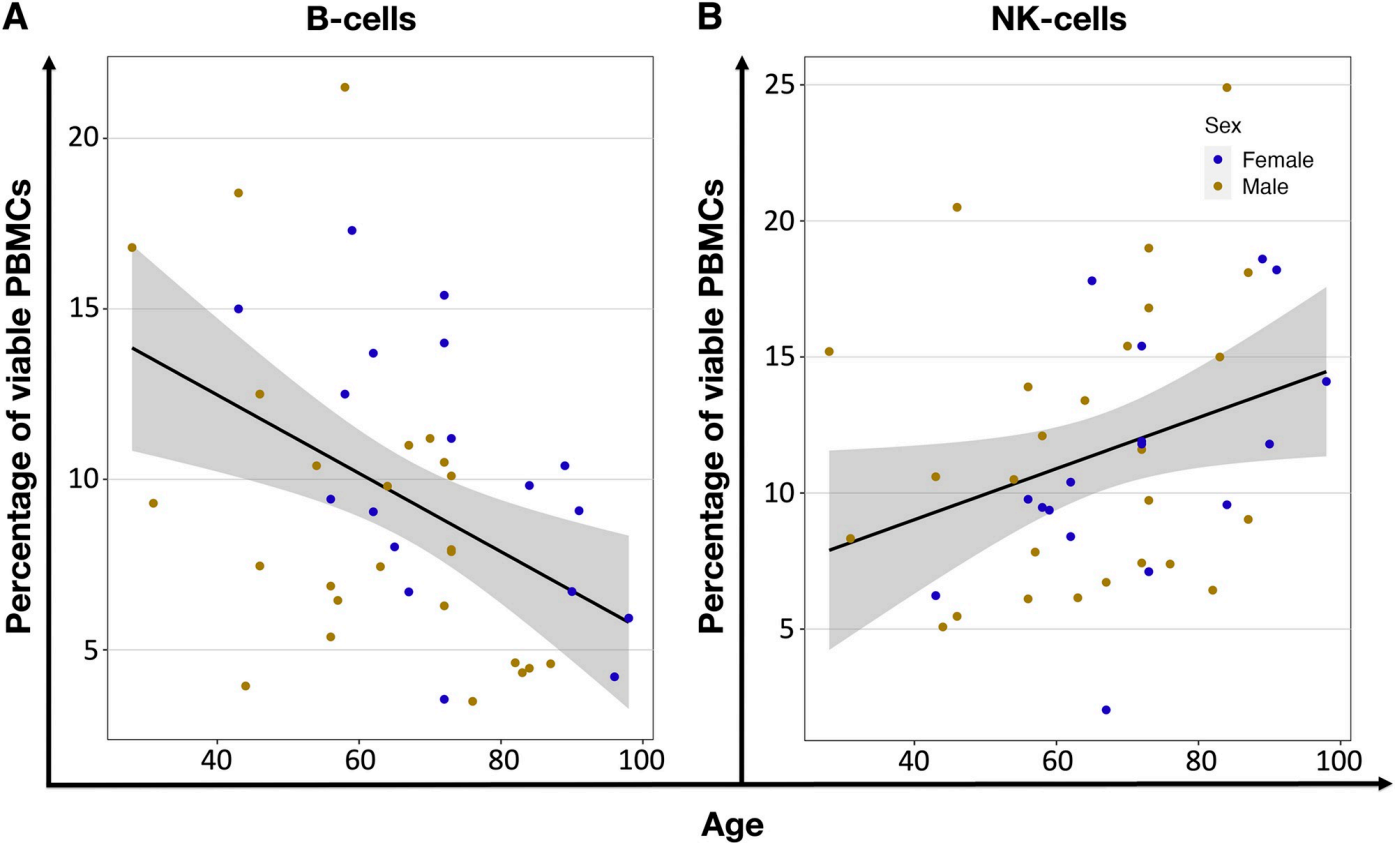
Age-related correlation of PBMCs percentages. **(A)** Shows the decrease (r = -0.44, P = 0.003) of B-cell, and (**B**) an increase (r = 0.31, P = 0.043) of NK-cell percentage within viable PBMCs. Each dot represents an individual donor, orange for males and blue for females. The black line shows linear regression, and the grey area is the 95% confidence interval.

We found no differences in PBMC population proportions when comparing the Adult and Older groups, suggesting that the overall distribution of these immune cells remains relatively stable with age. Adult donors had 11.1±4.8% B-cells, 21.6±9.8% NK-cells, 38.9± 8.2% T-cells, and 18.9± 6.8% monocytes. In the aged population, we found similar percentages: 10.9±14.6% B-cells, 29.3±15.2% NK-cells, 38.7±16.2% T-cells, and 19.9±10.6% monocytes (S1 Fig 1 in S1 File, S2 Table 5 in S2 File). When using Pearson’s linear regression method to correlate proportions of the different PBMCs with age, we found that while B-cells (P = 0.003) decreased, the NK-cells (P = 0.043) increased (Fig 3, S2 Table 7 in S2 File). This might suggest a shift towards innate immunity with age, as evidenced by the decline in adaptive immune cells like B-cells and the rise in NK-cells.

**Fig 4:**
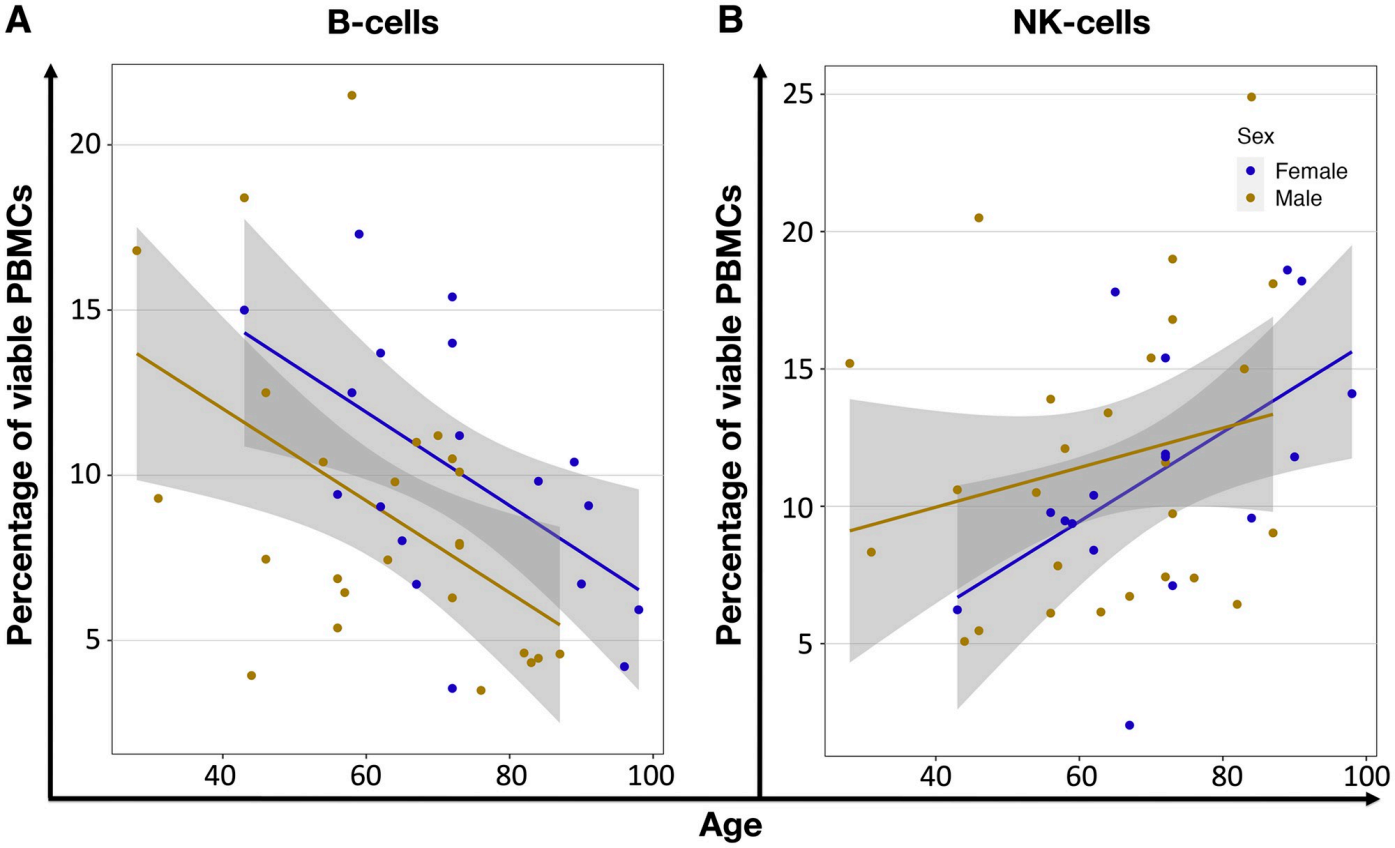
Age-related correlation of PBMCs percentages in comparison between males and females. Only cells/groups with significant p-values are shown. Additional data are presented in S1 Fig 5 in S1 File. (**A**) Shows the increased (r^f^ = 0.54, P = 0.025) proportion of female NK-cells and (**B**) decreased B-cells within viable PBMCs of female (r^f^ = -0.56, P = 0.016) and male (r^m^ = -0.49, P = 0.013) donors. Each circle represents an individual volunteer, orange for males and blue for females. The lines show the linear regression for each biological sex, and the grey area is the 95% confidence interval.

When analyzing the data separated by the donors’ sex, we detected a significant correlation between the female population’s age and the NK-cell percentage (P = 0.025). Additionally, we revealed a significant age-related decrease in B-cells within the viable female (P = 0.016) and male (P = 0.013) PBMCs (Fig 4, S2 Table 7 in S2 File).

The flow cytomery analysis of PBMCs shows that the proportions of B-cells and NK-cells vary significantly with age and sex. B-cells decrease with age, while NK-cells increase, indicating a shift towards innate immunity in older individuals. Furthermore, females show a significant correlation between age and NK-cell percentage, and both sexes experience a decrease in B-cells with age. These findings suggest notable sex-specific differences in immune aging, which could help explain sex disparities in immune function.

### Subtype quantification of peripheral blood monocytes

Subtype quantification of peripheral blood monocytes was conducted using flow cytometry analysis, which revealed that the vast majority of monocytes were represented by classical monocytes (CD14+/CD16-) (Fig 2C). They comprised 88.3±4.0% and 86.1±6.2% of all live monocytes in the Adult and Older groups, respectively. Intermediate (CD14+/CD16+) monocytes were 3.6±2.1% and 4.1±1.9% in the Adult and Older groups, and the non-classical (CD14-/CD16+) monocytes were 8.1±2.9% and 9.8±5.3% in the Adult and Older groups, correspondingly (S2 Table 5 in S2 File). Interestingly, no significant differences were observed in the proportions of these monocyte subtypes between the age groups (S1 Fig 1 in S1 File). The separate analysis, when groups were divided into males and females or correlated to age, did not reveal any age- or sex-dependent differences in the composition of monocytes either (S2 Table 5 in S2 File).

These findings underline the stability of monocyte subtype proportions across different demographic groups, suggesting that age and sex may not significantly influence the distribution of monocyte subsets in peripheral blood.

### Age-dependent expression of neuroinflammation- and regeneration-related genes in monocytes and their subtypes

**Fig 5:**
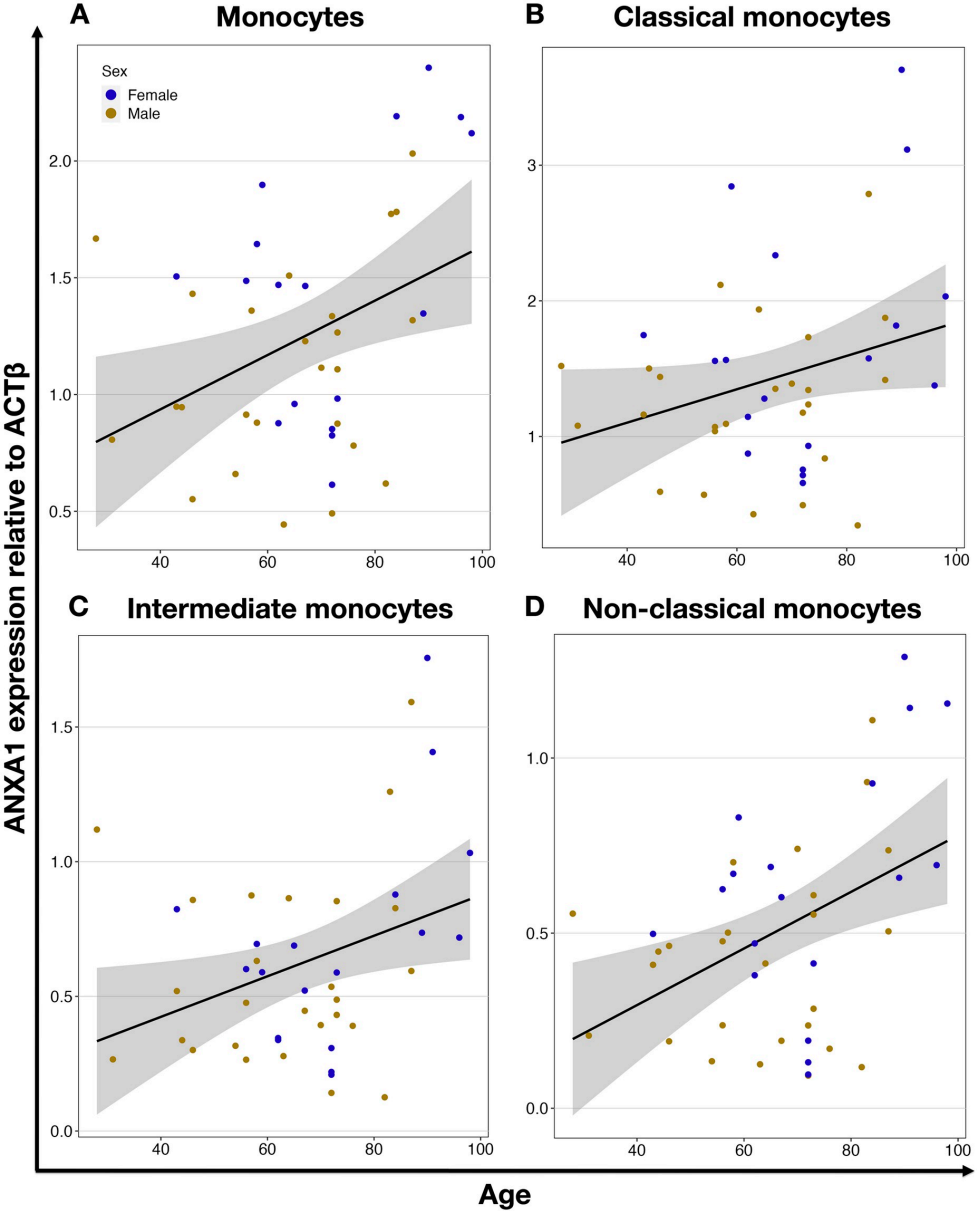
Age-dependent expression of *ANXA1* gene in monocytes and their subtypes. *ANXA1* expression in all monocytes (r = 0.38, P = 0.012) (**A**), classical (r = 0.28, P = 0.067) (**B**), intermediate (r = 0.33, P = 0.026) (**C**), and nonclassical (r = 0.43, P = 0.004) (**D**) monocytes. Each circle represents an individual donor, orange for males and blue for females. The black line shows linear regression, and the grey area is the 95% confidence interval.

Using the chip-based multiplex qPCR platform Fluidigm for the analysis of the expression of selected genes in monocytes, we revealed significant findings related to aging and gene expression. Specifically, we identified the anti-inflammatory gene annexin A1 (*ANXA1*, P = 0.012) and the pro-inflammatory scavenger receptor gene *CD36* (P = 0.042), which were significantly correlated to aging and both upregulated. Interestingly, the upregulation of the *ANXA1* gene was also observed in the intermediate (P = 0.026) and non-classical (P = 0.004) subtypes of the monocytes (Fig 5 and Table 1). This suggests that these monocyte subtypes may play a more prominent role in the anti-inflammatory response during aging, potentially contributing to the regulation of inflammation and tissue repair processes in the elderly. On the other hand, the age-related upregulation of *CD36* was not observed in any of the monocyte subpopulations (Table 1, S1 Table 8 in S1 File). This suggests that while *CD36* is upregulated in monocytes with age, this increase is not restricted to any particular subtype, and such widespread upregulation could indicate a general increase in pro-inflammatory activity within the aging monocyte population.

**Table 1:** Differentially expressed genes associated with age.

Monocyte subtype	Gene	Function	P value	N	Change	Regression coefficient	95% confidence interval	
**All**	*CD36*	Pro-inflammatory, scavenger receptor	0.042	44	↑	0.0019	0.0001	0.0038
**All**	*ANXA1*	Anti-inflammatory	0.012	43	↑	0.0116	0.0027	0.0206
**Classical**	*TLR8*	Pro-inflammatory	0.025	44	↓	- 0.0001	- 0.0003	- 1.8843
**Classical**	*S100A8*	Pro-inflammatory	0.022	44	↓	- 0.0317	- 0.0587	- 0.0047
**Intermediate**	*TNFα*	Pro-inflammatory	0.014	43	↓	- 0.0001	- 0.0002	- 0.0000
**Intermediate**	*TLR8*	Pro-inflammatory	0.008	42	↓	- 0.0002	- 0.0003	- 0.0001
**Intermediate**	*ANXA1*	Anti-inflammatory	0.026	44	↑	0.0075	0.0009	0.0141
**Intermediate**	*TGFβ1*	Anti-inflammatory	0.033	44	↑	0.0163	0.0014	0.0312
**Non-classical**	*TLR8*	Pro-inflammatory	0.014	44	↓	- 0.0001	- 0.0002	- 0.0001
**Non-classical**	*ANXA1*	Anti-inflammatory	0.004	44	↑	0.0081	0.0028	0.0134
**Non-classical**	*CD91*	Immune modulator	0.031	44	↑	0.0001	0,0000	0.0002

All genes showing significant changes in the expression level in primary human monocytes and their subtypes associated with age by Pearson correlation (P-value < 0.05). An upward arrow (↑) indicates increased gene expression, while a downward arrow (↓) indicates decreased gene expression. The regression coefficient and its corresponding 95% confidence interval quantify the relative increase in gene expression with each year of age.

### Age-dependent expression of other genes in different monocyte subtypes

We further analyzed the expression of selected monocyte-related genes in the three monocyte subtypes. Interestingly, even though there were no links between the expression of the chosen genes and the age of the donors when monocytes overall were studied, we detected several genes that were strongly correlated with the donors’ age but only in defined subtypes. Namely, in the classical monocyte subpopulation, we found the pro-inflammatory genes Toll-like receptor 8 (*TLR8*) (P = 0.025) and S100 calcium-binding protein A8 (*S100A8*) (P = 0.022) to be downregulated with increasing age. This suggests a potential reduction in the inflammatory response of classical monocytes as individuals age. This dual change could indicate a shift towards an anti-inflammatory state within intermediate monocytes, potentially playing a role in modulating age-related inflammation and maintaining tissue homeostasis. In the non-classical monocytes, we identified the immune modulator *CD91* (P = 0.031) as being upregulated with age. In the intermediate monocyte subpopulation, we additionally found that the anti-inflammatory gene *TGFβ1* (P = 0.033) was upregulated, and pro-inflammatory genes tumor necrosis factor *a* (*TNFa*, P = 0.014) and *TLR8* (P = 0.008) downregulated with increasing age. In the non-classical monocytes, we identified only the pro-inflammatory gene *TLR8* (P = 0.014) was downregulated and the immune modulator *CD91* (P = 0.031), was upregulated with aging (Table 1, S2 Table 8 in S2 File).

These findings highlight subtype-specific changes in gene expression associated with aging, suggesting a nuanced regulation of immune function within different monocyte populations. Such insights are crucial for understanding age-related alterations in immune responses and their potential implications for health and disease.

### Sex-dependent gene expression correlates with aging

**Fig 6:**
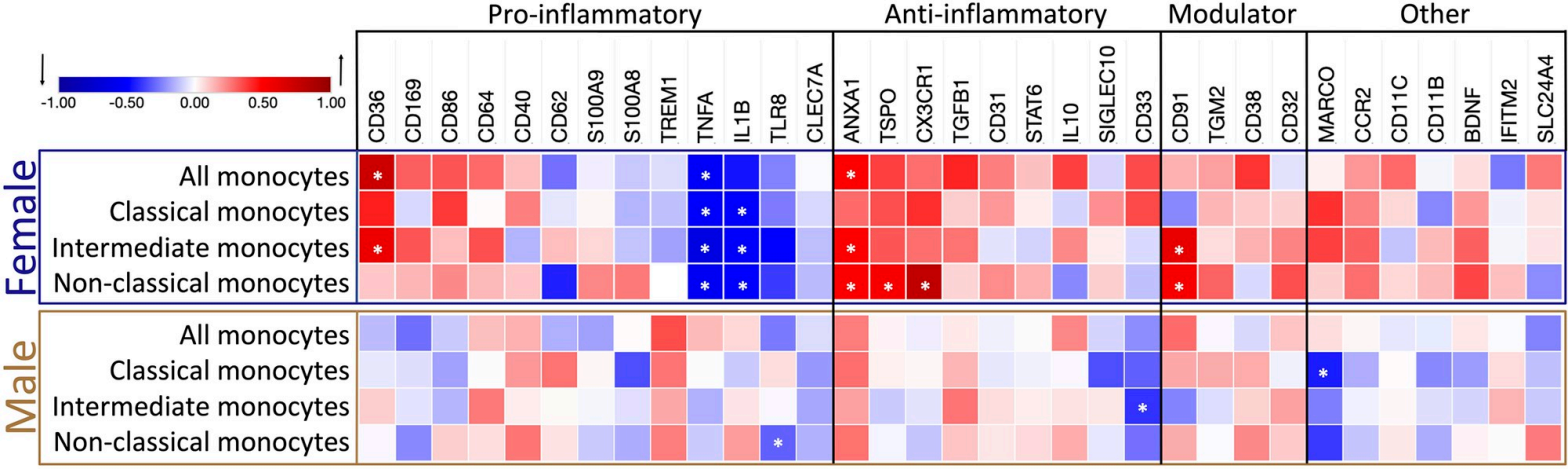
Heatmap of the relationship between age and gene expression in the different monocyte subtypes, separated by biological sex. The strength of the association of gene expression with age is represented by the Pearson correlation coefficient (r), ranging from -1, a perfect, negative correlation (blue); 0, no correlation; and 1, a perfect, positive correlation (red) of gene expression relative to *ACTβ* expression with increasing age. Genes are grouped by their most prevalent inflammatory function. Modulators have been linked to both pro- and anti-inflammatory functions. Female correlations are framed in blue (N = 18), and male correlations are in orange (N = 26). Significant genes, defined by a p-value below 0.05, are marked with an asterisk (*).

After revealing the age-dependent correlation in the expression of selected genes in monocyte subtypes, we further explored whether sex could be a factor for age-related changes (Fig 6).

Notably, most sex-driven changes in expression of the selected genes correlating with age were found in samples from female donors. Within the female population, age significantly impacted gene expression in overall monocytes. The anti-inflammatory gene *ANXA1* (P = 0.039) and pro-inflammatory *CD36* (P = 0.0003) were upregulated with age, while the pro-inflammatory gene *TNFα* (P = 0.024) was downregulated (Table 2).

**Table 2:** Age-dependent changes in neuroinflammation-related gene expression in males and females.

Monocyte subtype	Sex	Gene	Function	P value	N	Change	Regression coefficient (r)	95% confidence interval	
**All**	Female	*TNFα*	Pro-inflammatory	0.022	18	↓	- 0.0001	- 0.0002	0.0000
**All**	Female	*CD36*	Pro-inflammatory, scavenger receptor	0.0003	18	↑	0.0060	0.0032	0.0087
**All**	Female	*ANXA1*	Anti-inflammatory	0.039	18	↑	0.0246	0.0014	0.0478
**Classical**	Female	*TNFα*	Pro-inflammatory	0.035	17	↓	- 0.0001	- 0.0001	0.0000
**Classical**	Female	*IL-1β*	Pro-inflammatory	0.043	17	↓	- 0.0022	- 0.0044	- 0.0001
**Classical**	Male	*MARCO*	Phagocytotic activity, scavenger receptor	0.024	26	↓	- 0.0001	- 0.0002	- 0.0000
**Intermediate**	Female	*TNFα*	Pro-inflammatory	0.002	18	↓	- 0.0003	- 0.0004	- 0.0001
**Intermediate**	Female	*CD36*	Pro-inflammatory, scavenger receptor	0.010	18	↑	0.0027	0.0007	0.0046
**Intermediate**	Female	*IL-1β*	Pro-inflammatory	0.034	17	↓	- 0.0014	- 0.0027	- 0.0001
**Intermediate**	Female	*ANXA1*	Anti-inflammatory	0.038	18	↑	0.0127	0.0008	0.0246
**Intermediate**	Male	*CD33*	Anti-inflammatory	0.044	26	↓	- 0.0010	- 0.0019	- 0.0000
**Intermediate**	Female	*CD91*	Immune modulator	0.007	18	↑	0.0006	0.0002	0.0009
**Non-classical**	Female	*TNFα*	Pro-inflammatory	0.015	18	↓	- 0.0002	- 0.0003	- 0.0000
**Non-classical**	Female	*IL-1β*	Pro-inflammatory	0.038	18	↓	- 0.0004	- 0.0007	- 0.0000
**Non-classical**	Male	*TLR8*	Pro-inflammatory	0.024	26	↓	- 0.0003	- 0.0006	- 0.0000
**Non-classical**	Female	*CX3CR1*	Anti-inflammatory	0.0001	17	↑	0.0210	0.0130	0.0290
**Non-classical**	Female	*TSPO*	Anti-inflammatory	0.024	18	↑	0.0009	0,0001	0.0017
**Non-classical**	Female	*ANXA1*	Anti-inflammatory	0.035	18	↑	0.0110	0.0009	0.0211
**Non-classical**	Female	*CD91*	Immune modulator	0.023	18	↑	0.0002	0,0000	0.0003

The table presents only those genes that show an age-dependent significant change in expression level in primary human monocytes (males and females separately) and their subtypes (Pearson correlation; P-value < 0.05). An upward arrow (↑) indicates increased gene expression, while a downward arrow (↓) indicates decreased gene expression. The regression coefficient and its corresponding 95% confidence interval quantify the relative change in gene expression with each year of age.

When examining female monocyte subtypes, we observed that in classical monocytes, only the pro-inflammatory genes interleukin 1β (*IL-1β*, P = 0.043) and *TNFα* (P = 0.035) expressions were altered with aging, displaying a significant decrease, suggesting a reduction in inflammatory activity. In the intermediate subtype of female donors, three pro-inflammatory genes had significantly changed expression with age: *TNFα* (P = 0.002) and *IL-1β* (P = 0.034) were downregulated, and scavenger protein *CD36* (P = 0.01) was upregulated. The anti-inflammatory *ANXA1* (P = 0.038) and the immune modulator *CD91* (P = 0.007) were upregulated. In female non-classical monocytes, among the six genes that exhibited significant differential expression, all anti-inflammatory genes *ANXA1* (P = 0.035), *CX3CR1* (P = 0.0001), and translocator protein (*TSPO*, P = 0.024) showed upregulation, while the pro-inflammatory genes *IL-1β* (P = 0.038) and *TNFα* (P = 0.015) were downregulated with age. The immune modulator *CD91* (P = 0.023) is also upregulated in this subtype (Table 2). These findings demonstrate a clear trend of increased expression in the selected anti-inflammatory genes in female non-classical monocytes.

Moreover, using CD91 antibody for flow cytometry allowed us to analyze CD91 relative protein levels based on mean fluorescence intensity (MFI) in different monocyte subsets. Our study revealed that monocytes from older individuals did not show elevated CD91 MFI in any subgroup (S1 Fig 4 in S1 File). However, when we analyzed CD91 levels separately in monocytes from female and male donors, we observed that all monocyte subtypes in females exhibited increased CD91 MFI with aging. In contrast, no such correlation was found in male samples (S1 Fig 7 in S1 File). These protein-level results align with our gene expression data, further supporting our findings.

Only three genes in the monocyte subtype analysis were revealed as significantly downregulated in monocytes from male samples: in the classical subtype, the scavenger receptor macrophage receptor with collagenous structure (*MARCO*, P = 0.024), involved in phagocytotic activity; in the intermediate subtype, the anti-inflammatory gene *CD33* (P = 0.044); and in the non-classical subtype, the pro-inflammatory gene *TLR8* (P = 0.024) (Table 2, Fig 6, and S2 Table 8 in S2 File). These results highlight that sex-dependent gene expression correlates with aging, particularly emphasizing the more pronounced changes observed in females. The upregulation of anti-inflammatory genes and downregulation of pro-inflammatory genes in female monocytes suggest a complex regulatory mechanism that could contribute to sex-specific differences in immune aging.

## Discussion

Here, we have used flow cytometry, cell sorting, and high-throughput microfluidics-based quantitative PCR to determine how aging influences the expression of genes linked to neuroinflammation in subtypes of human peripheral blood monocytes. Our study, which included 26 male and 18 female healthy volunteers, revealed that in monocytes, genes associated with inflammation undergo progressive changes as individuals age, with the most significant alterations observed in females. The subtypes of monocytes differentially express inflammation-related genes. Some subtypes do not exhibit age-related changes in the expression of a given gene, while others show alterations of gene expression in either direction. However, we never observed age-related increased expression of a given gene in one subtype of monocytes and decreased expression in other subtypes or vice versa. It will be important to follow up on our study and verify the gene expression data by protein level or release. This can be achieved by applying relevant experimental approaches for different genes based on the nature of the protein they encode.

Our observations regarding age-related changes in the percentages of PBMCs were in accordance with previous reports: (i) The proportion of B- and T-cells and various monocyte subtypes was stable across age groups, with classical monocytes being the predominant population [14, 19]. (ii) We found age-related declines in the viability of peripheral blood mononuclear cells (PBMCs) as well as decreased percentage of B-cells, and an increased proportion of NK-cell populations. These findings align with existing literature [33–35]. (iii) When donors were separated by biological sex, the age-associated rise in NK-cell proportions was significant solely in females [36]. In contrast, B-cell counts were significantly decreased in both females and males [37]. We observed greater variability in immune cell counts in elderly individuals compared to younger participants. This most likely reflects the individual changes in the immune system that result from aging.

Seemingly at variance with the present data, Metcalf and co-workers [38] reported no significant age-related gene alterations in classical (CD14^+^CD16^-^), intermediate (CD14^+^CD16^+^), and non-classical (CD14^dim^CD16^+^) monocytes under non-stimulated conditions. In contrast, we detected alterations of several genes in all or subgroups of monocytes correlating with aging. The most likely explanation for this discrepancy is that Metcalf and co-workers divided the blood donors into two groups, adults and older subjects, with 11 individuals in each, and performed group comparison using 2-way ANOVA. They pooled both male and female data in a single adult and aged groups, respectively. When we divided the donors into two groups based on age (under and above 65 years), we found no significant differences in the proportions of different blood cells and monocyte subtypes, nor in the expression of several neuroinflammation- and regeneration-related genes between these groups. However, correlation analysis revealed age-related changes in cell percentages and gene expression when age was considered a continuous variable. This suggests that although specific age groups may not show strong differences, significant changes occur as individuals age continuously. This finding implies that each person has a unique aging curve for their immune system and monocyte subtypes expressing neuroinflammation- and regeneration-related genes. Introducing arbitrary age cut-offs to distinguish between adult and old populations may introduce bias and obscure the accurate picture based on individual variability [39]. Therefore, the Pearson correlation analysis of age-related changes offers more potential than separating data based on arbitrary age cut-offs, helping to reveal valuable insights into the dynamics of biological changes in aging individuals.

Ischemic stroke ranks among the leading causes of death and disability, with its prevalence increasing with age, and the biological sex of the patients influencing it. Neuroinflammation is a crucial factor in this disease, where immune cells, including monocytes, initially contribute to brain damage but later aid in tissue regeneration and functional recovery. In our previous studies [4, 11], we have shown the important role of MDMs in post-stroke recovery. Therefore, we decided in the present study to examine and discuss how age and sex influence the expression of genes linked to neuroinflammation and regeneration in monocytes and their subtypes. However, we are aware that observed age- and sex-related changes in the monocyte gene expression might be of importance to incidence, severity, and prognosis for other diseases.

In our study, for the first time, we demonstrated increased expression of the anti-inflammatory *ANXA1* gene in the CD91+ population of monocytes, driven by higher levels of this gene in intermediate and non-classical subtypes of monocytes in relation to aging. *ANXA1* is a gene coding for Annexin A1 [40], a protein that exerts anti-inflammatory effects through the G-coupled formyl peptide receptor type-2 with a phospholipase A2 inhibitory activity (reviewed by Perretti and D’Acquisto [41]).

While the involvement of *ANXA1* in regulating inflammation is well-documented, the relevance of *ANXA1* expression in longevity and healthy aging is unknown, and our finding implicates its potential connection with the aging process. Notably, *ANXA1* has been demonstrated to be involved in a variety of biological processes, such as the regulation of macrophage phagocytosis and neutrophil migration, acute [42] and chronic [43] inflammation, and ischemia/reperfusion injuries [42, 44, 45]. The restoration of plasma *ANXA1* levels after stroke is indicative of a favorable recovery in stroke patients, suggesting its potential as a biomarker and valuable prognostic tool [46]. It has been shown that the classical monocyte subtype conveys detrimental effects after stroke, including stronger interaction with platelets [12]. In contrast, non-classical and intermediate monocytes are beneficial with a phenotype that could promote tissue repair and angiogenesis. The negative outcome after stroke is increased with aging [47], and the lack of the increase of *ANXA1* expression in classical monocytes might be a confounding factor.

We detected increased expression of the pro-inflammatory *CD36* gene in all monocytes with aging. *CD36*, a multi-ligand scavenger receptor, manifests a robust pro-inflammatory response when expressed in monocytes and macrophages. *CD36* mediates innate immunity, participating in the assembly of inflammatory pathways and contributing to reactive oxygen species (ROS) production [48], and plays a role in macrophage phagocytosis during the resolution phase of ischemic stroke in mice [49].

Inflammation is vital in maintaining the body’s homeostasis and promoting recovery after injury. However, if inflammation becomes excessively aggressive or persists without being resolved, it can result in profound tissue damage [50]. The observed upregulation of the *CD36* gene in all monocytes suggests a potential shift towards a more pro-inflammatory phenotype during aging. In contrast, the concurrent upregulation of the *ANXA1* gene expression suggests an anti-inflammatory tendency. Therefore, it is plausible that the increased expression of *ANXA1* and *CD36* may act in concert, potentially offsetting each other and pointing towards dysregulation in inflammation mechanisms during aging.

We detected the downregulation of the *S100A8* gene, a calcium-binding protein belonging to the S100 family [51] only in classical monocytes. The S100a8 (calgranulin A) and S100a9 (calgranulin B) proteins are constitutively expressed in neutrophils and monocytes. They are potential biomarkers for inflammation-associated diseases and key inflammatory regulators with the capacity to initiate and react to signals associated with inflammation [52]. In contrast with our findings, it has been shown that the increase in expression of *S100A8/A9*, particularly *S100A9*, represents a characteristic of aging across various mammalian tissues [53]. This phenomenon involves diverse cell types, including those in the blood and the central nervous system. In healthy human donors, decreased levels of the *S100A8/S100A9* were found in the serum of the elderly individuals compared to the young. However, data regarding the expression of *S100A8* in monocytes with aging are not entirely consistent across studies. It has been shown that the expression of *S100A8* in classical and intermediate monocytes individually is higher compared to the other two subtypes, but in non-classical, it is lower [38]. Moreover, *S100A8* also differentially responds to the activation of monocyte subtypes, though with similar changes in cells from young and old donors [38]. It is important to note that such discrepancy could be attributed to the fact that these studies did not specifically focus on monocytes; instead, they analyzed whole blood or other tissues. Currently, aging is often associated with increased pro-inflammatory cytokines in blood plasma. However, our data underscores the importance of discerning the distinct contributions of various monocyte classes to the aging phenomenon.

In the intermediate monocytes, in addition to *ANXA1*, the anti-inflammatory *TGFβ1* gene was upregulated, and the pro-inflammatory genes *TNFα* and *TLR8* were downregulated with increasing age. Although intermediate monocytes act as antigen-presenting cells, secrete cytokines, and regulate apoptosis, their precise role in immunity appears elusive [54]. The concomitant age-dependent anti- and pro-inflammatory gene expression changes suggest a potential contribution to immune dysregulation and inflammation of the intermediate monocytes.

In non-classical monocytes, two genes, *ANXA1* and *CD91*, were upregulated with aging, while *TLR8* was downregulated. In the present study, we have successfully used CD91, the adhesion molecule, for the identification of human monocytes in a more accurate manner instead of solely relying on the CD14 and CD16 expression [29]. CD91 has been identified as a receptor in antigen-presenting cells, including monocytes, which plays a crucial role in the innate and adaptive immune response [55]. In a recent study, it was shown that expression of CD91detected by FACS on different monocyte subsets was one of the best predictors of age advancement in a combined population consisting of people with HIV, HIV-negative, and blood donors [56]. In the present study, we also demonstrated at gene expression and protein levels that CD91 is upregulated with aging in all monocyte subtypes but only in females, not males. These results align with our other findings that inflammation-related genes are differentially correlated with aging in different monocyte subsets, predominantly in females.

After revealing the age-dependent correlation of gene expression in monocyte subtypes, we further explored whether biological sex could contribute to the observed age-related changes. Classical monocytes isolated from male donors show downregulation of *MARCO* and intermediate monocytes downregulated the anti-inflammatory marker *CD33*. Notably, the female donors display changes in the phenotype of all three monocyte subtypes with age. At the same time, the classical monocytes showed downregulation, specifically of pro-inflammatory genes *TNFα* and *IL-1β*. The intermediate and non-classical subclasses exhibited significant alterations in the expression of multiple genes, indicating the acquisition of an ambiguous pro- and anti-inflammatory phenotype with aging.

We report for the first time a significant downregulation of the *TLR8* gene expression in non-classical monocytes of male donors with aging. *TLR7* and *TLR8* are crucial components of the innate immune response, recognizing RNA degradation products from pathogens. *TLR8*, found in monocytes and dendritic cells, is not subject to X chromosome inactivation in certain cells, possibly leading to higher *TLR8* levels in females. This has implications for antiviral and antibacterial responses, as well as susceptibility to inflammatory and autoimmune diseases, highlighting *TLR8*’s role in immune regulation and disease vulnerability [57]. In a recent study, TLR8 protein expression was reported to be higher in all subsets of female monocytes compared to their male counterparts in healthy blood donors aged 16–44 years, and flow cytometry analysis revealed that, on average, 60% of non-classical monocytes positively stained for TLR8 [58]. Intriguingly, this suggests a potential alteration in pathogen response and increased susceptibility to autoimmune and inflammatory diseases in aging individuals.

We found that the *TSPO* gene, encoding the mitochondrial membrane protein, is strongly upregulated with aging, exclusively in non-classical monocytes of female donors. TSPO is found in diverse cell types, including brain microglia cells and circulating lymphocytes and monocytes [59]. Previous *ex-vivo* human studies demonstrated the impact of various TSPO ligands on the monocyte chemotaxis [60]. Recently, it has been hypothesized that peripheral monocytes’ recruitment through the blood-brain barrier may contribute to the increase of TSPO levels in the central nervous system observed in the context of inflammation and Alzheimer’s disease [61]. Our findings reveal a novel association between age, *TSPO* gene expression, and the unique functional characteristics of non-classical monocytes. The upregulation of *TSPO* in female donors could play a role in highly specialized functions of non-classical monocytes, which include trans-endothelial migration, phagocytosis, and viral response, and may contribute to the overall immune surveillance in aging individuals.

Aging exhibits sex-specificity, and in women, it is characterized by the occurrence of menopause, typically taking place between 45 and 55 years of age globally (World Health Organization, 2022). Notably, our study shows that female donors display robust age-related changes in the phenotypes of all three monocyte subtypes. Our study’s age range of female donors extends from 43 to 98 years, suggesting a potential overrepresentation of peri-menopausal and menopausal women within the female sample group. Previous studies have shown disruptions in the cyclic pattern of circulating estrogen, a potent anti-inflammatory agent, [62] during the menopausal shift. This activates broader innate and adaptive immune reactions in the body [63], elevating levels of chronic systemic inflammation [64], increasing the risk of cardiovascular diseases [65], and making the brain more susceptible to ischemic damage. This highlights the potential complex interplay of monocyte subtypes in the context of aging.

In a previously published study exclusively examining females comparing pre-menopausal (50.0 ± 3.1 years old) and post-menopausal (52.0 ± 1.7 years old) women, it was noted that menopause influences the functional state of circulating monocytes [66]. Our study extends this knowledge, demonstrating that females exhibit more pronounced changes in gene expression among monocyte subtypes than males, emphasizing the significant impact on females in the context of aging. Consequently, comprehending the biological mechanisms underlying the transition to menopause can contribute to the development of strategies aimed at protecting women from health complications associated with menopause.

In conclusion, our data show that the expression levels of several inflammation- and regeneration-related genes in human monocyte subtypes change with aging, predominantly in females. This highlights the importance of age consideration for patients with inflammation-related diseases. Moreover, the monocyte-related inflammatory response should probably be considered from a monocyte subtype perspective. Our data suggest that, in the light of personalized medicine, biological sex should be considered as a factor when designing individual treatments for different inflammation-linked and -causing diseases. Future research that expands beyond healthy volunteers and focuses on comprehensive gene studies in patients is highly warranted.

## Supporting information

S1 File Supplementary Figs 1–7. (1) Flow cytometry analysis of PBMCs and the subpopulations of monocytes. (2) Age-related correlation of PBMCs proportions. (3) Age-related correlation of monocyte subtype proportions. (4) Age-related correlation of mean fluorescence intensity (MFI) of CD91 in monocyte subtypes. (5) Age-related correlation of PBMCs proportions analyzed separately for males and females. (6) Age-related correlation of monocyte subtype proportions analyzed separately for males and females. (7) Age-related correlation of mean fluorescence intensity (MFI) of CD91 in monocyte subtypes analyzed separately for males and females.
(PPTX)

S2 File Supplementary Tables 1–8. (1) Metadata of patient samples. (2) List of flow cytomery antibodies used in this study. (3) Flow cytomery antibody MasterMix per sample / PBMCs. (4) Flow cytomery analysis results. (5) Row statistics of flow cytomery analysis—Adult vs Older. (6) List of primers used in the study. (7) Linear regression analysis (Pearson) for cell populations with age. (8) Linear regression analysis (Pearson) for gene expressions with age.
(XLSX)

## References

[1] Franceschi C, Garagnani P, Parini P, Giuliani C, Santoro A. Inflammaging: a new immune-metabolic viewpoint for age-related diseases. Nat Rev Endocrinol. 2018;14(10):576–90. doi: 10.1038/s41574-018-0059-4 30046148

[2] Wynn TA, Chawla A, Pollard JW. Macrophage biology in development, homeostasis and disease. Nature. 2013;496(7446):445–55. doi: 10.1038/nature12034 23619691 PMC3725458

[3] Moss CE, Johnston SA, Kimble JV, Clements M, Codd V, Hamby S, et al. Aging-related defects in macrophage function are driven by MYC and USF1 transcriptional programs. Cell Rep. 2024;43(4):114073. doi: 10.1016/j.celrep.2024.114073 38578825

[4] Wattananit S, Tornero D, Graubardt N, Memanishvili T, Monni E, Tatarishvili J, et al. Monocyte-Derived Macrophages Contribute to Spontaneous Long-Term Functional Recovery after Stroke in Mice. J Neurosci. 2016;36(15):4182–95. doi: 10.1523/JNEUROSCI.4317-15.2016 27076418 PMC6601783

[5] Grage-Griebenow E, Flad HD, Ernst M. Heterogeneity of human peripheral blood monocyte subsets. J Leukoc Biol. 2001;69(1):11–20. doi: 10.1182/blood.V74.7.2527.2527 11200054

[6] Gordon S. Alternative activation of macrophages. Nat Rev Immunol. 2003;3(1):23–35. doi: 10.1038/nri978 12511873

[7] Zera KA, Buckwalter MS. The Local and Peripheral Immune Responses to Stroke: Implications for Therapeutic Development. Neurotherapeutics. 2020;17(2):414–35. doi: 10.1007/s13311-020-00844-3 32193840 PMC7283378

[8] Jayaraj RL, Azimullah S, Beiram R, Jalal FY, Rosenberg GA. Neuroinflammation: friend and foe for ischemic stroke. J Neuroinflammation. 2019;16(1):142. doi: 10.1186/s12974-019-1516-2 31291966 PMC6617684

[9] Var SR, Shetty AV, Grande AW, Low WC, Cheeran MC. Microglia and Macrophages in Neuroprotection, Neurogenesis, and Emerging Therapies for Stroke. Cells. 2021;10(12):3555. doi: 10.3390/cells10123555 34944064 PMC8700390

[10] Wicks EE, Ran KR, Kim JE, Xu R, Lee RP, Jackson CM. The Translational Potential of Microglia and Monocyte-Derived Macrophages in Ischemic Stroke. Front Immunol. 2022;13:897022. doi: 10.3389/fimmu.2022.897022 35795678 PMC9251541

[11] Ge R, Tornero D, Hirota M, Monni E, Laterza C, Lindvall O, et al. Choroid plexus-cerebrospinal fluid route for monocyte-derived macrophages after stroke. J Neuroinflammation. 2017;14(1):153. doi: 10.1186/s12974-017-0909-3 28754163 PMC5534106

[12] Urra X, Villamor N, Amaro S, Gomez-Choco M, Obach V, Oleaga L, et al. Monocyte subtypes predict clinical course and prognosis in human stroke. J Cereb Blood Flow Metab. 2009;29(5):994–1002. doi: 10.1038/jcbfm.2009.25 19293821

[13] Ziegler-Heitbrock L, Ancuta P, Crowe S, Dalod M, Grau V, Hart DN, et al. Nomenclature of monocytes and dendritic cells in blood. Blood. 2010;116(16):e74–80. doi: 10.1182/blood-2010-02-258558 20628149

[14] Thomas GD, Hamers AAJ, Nakao C, Marcovecchio P, Taylor AM, McSkimming C, et al. Human Blood Monocyte Subsets: A New Gating Strategy Defined Using Cell Surface Markers Identified by Mass Cytometry. Arterioscler Thromb Vasc Biol. 2017;37(8):1548–58. doi: 10.1161/ATVBAHA.117.309145 28596372 PMC5828170

[15] Cormican S, Griffin MD. Human Monocyte Subset Distinctions and Function: Insights From Gene Expression Analysis. Front Immunol. 2020;11:1070. doi: 10.3389/fimmu.2020.01070 32582174 PMC7287163

[16] Kapellos TS, Bonaguro L, Gemund I, Reusch N, Saglam A, Hinkley ER, et al. Human Monocyte Subsets and Phenotypes in Major Chronic Inflammatory Diseases. Front Immunol. 2019;10:2035. doi: 10.3389/fimmu.2019.02035 31543877 PMC6728754

[17] Guilliams M, Mildner A, Yona S. Developmental and Functional Heterogeneity of Monocytes. Immunity. 2018;49(4):595–613. doi: 10.1016/j.immuni.2018.10.005 . 30332628

[18] Hudig D, Hunter KW, Diamond WJ, Redelman D. Properties of human blood monocytes. II. Monocytes from healthy adults are highly heterogeneous within and among individuals. Cytometry B Clin Cytom. 2014;86(2):121–34. doi: 10.1002/cyto.b.21141 24327358 PMC4854626

[19] Ozanska A, Szymczak D, Rybka J. Pattern of human monocyte subpopulations in health and disease. Scand J Immunol. 2020;92(1):e12883. doi: 10.1111/sji.12883 32243617

[20] Marino Lee S, Hudobenko J, McCullough LD, Chauhan A. Microglia depletion increase brain injury after acute ischemic stroke in aged mice. Exp Neurol. 2021;336:113530. doi: 10.1016/j.expneurol.2020.113530 33221396 PMC7856174

[21] Spychala MS, Honarpisheh P, McCullough LD. Sex differences in neuroinflammation and neuroprotection in ischemic stroke. J Neurosci Res. 2017;95(1–2):462–71. doi: 10.1002/jnr.23962 27870410 PMC5217708

[22] Seidler S, Zimmermann HW, Bartneck M, Trautwein C, Tacke F. Age-dependent alterations of monocyte subsets and monocyte-related chemokine pathways in healthy adults. BMC Immunol. 2010;11:30. doi: 10.1186/1471-2172-11-30 20565954 PMC2910032

[23] Saare M, Tserel L, Haljasmagi L, Taalberg E, Peet N, Eimre M, et al. Monocytes present age-related changes in phospholipid concentration and decreased energy metabolism. Aging Cell. 2020;19(4):e13127. doi: 10.1111/acel.13127 32107839 PMC7189998

[24] So J, Tai AK, Lichtenstein AH, Wu D, Lamon-Fava S. Sexual dimorphism of monocyte transcriptome in individuals with chronic low-grade inflammation. Biol Sex Differ. 2021;12(1):43. doi: 10.1186/s13293-021-00387-y 34321081 PMC8320037

[25] Varghese M, Clemente J, Lerner A, Abrishami S, Islam M, Subbaiah P, et al. Monocyte Trafficking and Polarization Contribute to Sex Differences in Meta-Inflammation. Front Endocrinol (Lausanne). 2022;13:826320. doi: 10.3389/fendo.2022.826320 35422759 PMC9001155

[26] Appleby LJ, Nausch N, Midzi N, Mduluza T, Allen JE, Mutapi F. Sources of heterogeneity in human monocyte subsets. Immunol Lett. 2013;152(1):32–41. doi: 10.1016/j.imlet.2013.03.004 23557598 PMC3684771

[27] Patel AA, Yona S. Inherited and Environmental Factors Influence Human Monocyte Heterogeneity. Front Immunol. 2019;10:2581. doi: 10.3389/fimmu.2019.02581 31787976 PMC6854020

[28] Yoon CW, Bushnell CD. Stroke in Women: A Review Focused on Epidemiology, Risk Factors, and Outcomes. J Stroke. 2023;25(1):2–15. doi: 10.5853/jos.2022.03468 36746378 PMC9911842

[29] Hudig D, Hunter KW, Diamond WJ, Redelman D. Properties of human blood monocytes. I. CD91 expression and log orthogonal light scatter provide a robust method to identify monocytes that is more accurate than CD14 expression. Cytometry B Clin Cytom. 2014;86(2):111–20. doi: 10.1002/cyto.b.21131 24591168 PMC4854625

[30] Sanchez-Freire V, Ebert AD, Kalisky T, Quake SR, Wu JC. Microfluidic single-cell real-time PCR for comparative analysis of gene expression patterns. Nat Protoc. 2012;7(5):829–38. doi: 10.1038/nprot.2012.021 22481529 PMC3657501

[31] McCall MN, McMurray HR, Land H, Almudevar A. On non-detects in qPCR data. Bioinformatics. 2014;30(16):2310–6. doi: 10.1093/bioinformatics/btu239 24764462 PMC4133581

[32] Li P, Stuart EA, Allison DB. Multiple Imputation: A Flexible Tool for Handling Missing Data. JAMA. 2015;314(18):1966–7. doi: 10.1001/jama.2015.15281 26547468 PMC4638176

[33] Le Garff-Tavernier M, Beziat V, Decocq J, Siguret V, Gandjbakhch F, Pautas E, et al. Human NK cells display major phenotypic and functional changes over the life span. Aging Cell. 2010;9(4):527–35. doi: 10.1111/j.1474-9726.2010.00584.x 20477761

[34] Frasca D, Blomberg BB. Aging impairs murine B cell differentiation and function in primary and secondary lymphoid tissues. Aging Dis. 2011;2(5):361–73 22396888 PMC3295082

[35] Gounder SS, Abdullah BJJ, Radzuanb N, Zain F, Sait NBM, Chua C, et al. Effect of Aging on NK Cell Population and Their Proliferation at Ex Vivo Culture Condition. Anal Cell Pathol (Amst). 2018;2018:7871814. doi: 10.1155/2018/7871814 30175033 PMC6098903

[36] Hirokawa K, Utsuyama M, Hayashi Y, Kitagawa M, Makinodan T, Fulop T. Slower immune system aging in women versus men in the Japanese population. Immun Ageing. 2013;10(1):19. doi: 10.1186/1742-4933-10-19 23675689 PMC3663722

[37] Wikby A, Johansson B, Ferguson F, Olsson J. Age-related changes in immune parameters in a very old population of Swedish people: a longitudinal study. Exp Gerontol. 1994;29(5):531–41. doi: 10.1016/0531-5565(94)90036-1 7828662

[38] Metcalf TU, Wilkinson PA, Cameron MJ, Ghneim K, Chiang C, Wertheimer AM, et al. Human Monocyte Subsets Are Transcriptionally and Functionally Altered in Aging in Response to Pattern Recognition Receptor Agonists. J Immunol. 2017;199(4):1405–17. doi: 10.4049/jimmunol.1700148 28696254 PMC5548610

[39] Montgomery DC, Peck E.A., and Vining G.G. Introduction to Linear Regression Analysis. 5th ed: Wiley-Blackwell; 2021.

[40] Lim LH, Pervaiz S. Annexin 1: the new face of an old molecule. FASEB J. 2007;21(4):968–75. doi: 10.1096/fj.06-7464rev 17215481

[41] Perretti M, D’Acquisto F. Annexin A1 and glucocorticoids as effectors of the resolution of inflammation. Nat Rev Immunol. 2009;9(1):62–70. doi: 10.1038/nri2470 19104500

[42] Gavins FN, Yona S, Kamal AM, Flower RJ, Perretti M. Leukocyte antiadhesive actions of annexin 1: ALXR- and FPR-related anti-inflammatory mechanisms. Blood. 2003;101(10):4140–7. doi: 10.1182/blood-2002-11-3411 12560218

[43] Goulding NJ, Euzger HS, Butt SK, Perretti M. Novel pathways for glucocorticoid effects on neutrophils in chronic inflammation. Inflamm Res. 1998;47 Suppl 3:S158–65. doi: 10.1007/s000110050310 9831319

[44] D’Amico M, Di Filippo C, La M, Solito E, McLean PG, Flower RJ, et al. Lipocortin 1 reduces myocardial ischemia-reperfusion injury by affecting local leukocyte recruitment. FASEB J. 2000;14(13):1867–9. doi: 10.1096/fj.99-0602fje 11023969

[45] La M, D’Amico M, Bandiera S, Di Filippo C, Oliani SM, Gavins FN, et al. Annexin 1 peptides protect against experimental myocardial ischemia-reperfusion: analysis of their mechanism of action. FASEB J. 2001;15(12):2247–56. doi: 10.1096/fj.01-0196com 11641252

[46] Xu X, Gao W, Li L, Hao J, Yang B, Wang T, et al. Annexin A1 protects against cerebral ischemia-reperfusion injury by modulating microglia/macrophage polarization via FPR2/ALX-dependent AMPK-mTOR pathway. J Neuroinflammation. 2021;18(1):119. doi: 10.1186/s12974-021-02174-3 34022892 PMC8140477

[47] Ritzel RM, Lai YJ, Crapser JD, Patel AR, Schrecengost A, Grenier JM, et al. Aging alters the immunological response to ischemic stroke. Acta Neuropathol. 2018;136(1):89–110. doi: 10.1007/s00401-018-1859-2 29752550 PMC6015099

[48] Cho S, Park EM, Febbraio M, Anrather J, Park L, Racchumi G, et al. The class B scavenger receptor CD36 mediates free radical production and tissue injury in cerebral ischemia. J Neurosci. 2005;25(10):2504–12. doi: 10.1523/JNEUROSCI.0035-05.2005 15758158 PMC6725161

[49] Woo MS, Yang J, Beltran C, Cho S. Cell Surface CD36 Protein in Monocyte/Macrophage Contributes to Phagocytosis during the Resolution Phase of Ischemic Stroke in Mice. J Biol Chem. 2016;291(45):23654–61. doi: 10.1074/jbc.M116.750018 27646002 PMC5095418

[50] Serhan CN, Chiang N. Endogenous pro-resolving and anti-inflammatory lipid mediators: a new pharmacologic genus. Br J Pharmacol. 2008;153 Suppl 1(Suppl 1):S200–15. doi: 10.1038/sj.bjp.0707489 17965751 PMC2268040

[51] Roth J, Vogl T, Sorg C, Sunderkotter C. Phagocyte-specific S100 proteins: a novel group of proinflammatory molecules. Trends Immunol. 2003;24(4):155–8. doi: 10.1016/s1471-4906(03)00062-0 12697438

[52] Wang S, Song R, Wang Z, Jing Z, Wang S, Ma J. S100A8/A9 in Inflammation. Front Immunol. 2018;9:1298. doi: 10.3389/fimmu.2018.01298 29942307 PMC6004386

[53] Swindell WR, Johnston A, Xing X, Little A, Robichaud P, Voorhees JJ, et al. Robust shifts in S100a9 expression with aging: a novel mechanism for chronic inflammation. Sci Rep. 2013;3:1215. doi: 10.1038/srep01215 23386971 PMC3564041

[54] Dally S, Lemuth K, Kaase M, Rupp S, Knabbe C, Weile J. DNA microarray for genotyping antibiotic resistance determinants in Acinetobacter baumannii clinical isolates. Antimicrob Agents Chemother. 2013;57(10):4761–8. doi: 10.1128/AAC.00863-13 23856783 PMC3811405

[55] Basu S, Binder RJ, Ramalingam T, Srivastava PK. CD91 is a common receptor for heat shock proteins gp96, hsp90, hsp70, and calreticulin. Immunity. 2001;14(3):303–13. doi: 10.1016/s1074-7613(01)00111-x 11290339

[56] De Francesco D, Sabin CA, Reiss P, Kootstra NA. Monocyte and T Cell Immune Phenotypic Profiles Associated With Age Advancement Differ Between People With HIV, Lifestyle-Comparable Controls and Blood Donors. Front Immunol. 2020;11:581616. doi: 10.3389/fimmu.2020.581616 33123168 PMC7573236

[57] Heil F, Hemmi H, Hochrein H, Ampenberger F, Kirschning C, Akira S, et al. Species-specific recognition of single-stranded RNA via toll-like receptor 7 and 8. Science. 2004;303(5663):1526–9. doi: 10.1126/science.1093620 14976262

[58] Youness A, Cenac C, Faz-Lopez B, Grunenwald S, Barrat FJ, Chaumeil J, et al. TLR8 escapes X chromosome inactivation in human monocytes and CD4(+) T cells. Biol Sex Differ. 2023;14(1):60. doi: 10.1186/s13293-023-00544-5 37723501 PMC10506212

[59] Woods MJ, Williams DC. Multiple forms and locations for the peripheral-type benzodiazepine receptor. Biochem Pharmacol. 1996;52(12):1805–14. doi: 10.1016/s0006-2952(96)00558-8 8951338

[60] Ferrarese C, Appollonio I, Frigo M, Perego M, Pierpaoli C, Trabucchi M, et al. Characterization of peripheral benzodiazepine receptors in human blood mononuclear cells. Neuropharmacology. 1990;29(4):375–8. doi: 10.1016/0028-3908(90)90097-b 2160625

[61] Conti E, Grana D, Angiulli F, Karantzoulis A, Villa C, Combi R, et al. TSPO Modulates Oligomeric Amyloid-beta-Induced Monocyte Chemotaxis: Relevance for Neuroinflammation in Alzheimer’s Disease. J Alzheimers Dis. 2023;95(2):549–59. doi: 10.3233/JAD-230239 37574731

[62] Vegeto E, Benedusi V, Maggi A. Estrogen anti-inflammatory activity in brain: a therapeutic opportunity for menopause and neurodegenerative diseases. Front Neuroendocrinol. 2008;29(4):507–19. doi: 10.1016/j.yfrne.2008.04.001 18522863 PMC2630539

[63] Giannoni E, Guignard L, Knaup Reymond M, Perreau M, Roth-Kleiner M, Calandra T, et al. Estradiol and progesterone strongly inhibit the innate immune response of mononuclear cells in newborns. Infect Immun. 2011;79(7):2690–8. doi: 10.1128/IAI.00076-11 21518785 PMC3191988

[64] Abildgaard J, Tingstedt J, Zhao Y, Hartling HJ, Pedersen AT, Lindegaard B, et al. Increased systemic inflammation and altered distribution of T-cell subsets in postmenopausal women. PLoS One. 2020;15(6):e0235174. doi: 10.1371/journal.pone.0235174 32574226 PMC7310708

[65] Ryczkowska K, Adach W, Janikowski K, Banach M, Bielecka-Dabrowa A. Menopause and women’s cardiovascular health: is it really an obvious relationship? Archives of Medical Science. 2023;19(2):458–66. doi: 10.5114/aoms/157308 37034510 PMC10074318

[66] Dvornyk V, Liu Y, Lu Y, Shen H, Lappe JM, Lei S, et al. Effect of menopause on gene expression profiles of circulating monocytes: a pilot in vivo microarray study. J Genet Genomics. 2007;34(11):974–83. doi: 10.1016/S1673-8527(07)60110-6 18037134

