# Supplementary material for: Human monocyte subtype expression of neuroinflammation- and regeneration-related genes is linked to age and sex
Source: PLoS One. 2024 Oct 30;19(10):e0300946. doi: 10.1371/journal.pone.0300946 (PMC11524521; doi:10.1371/journal.pone.0300946)
Supplement: S1 File — (1) Flow cytometry analysis of PBMCs and the subpopulations of monocytes. (2) Age-related correlation of PBMCs proportions. (3) Age-related correlation of monocyte subtype proportions. (4) Age-related correlation of mean fluorescence intensity (MFI) of CD91 in monocyte subtypes. (5) Age-related correlation of PBMCs proportions analyzed separately for males and females. (6) Age-related correlation of monocyte subtype proportions analyzed separately for males and females. (7) Age-related correlation of mean fluorescence intensity (MFI) of CD91 in monocyte subtypes analyzed separately for males and females. (PPTX) [file pone.0300946.s001.pptx]

## Slide 1
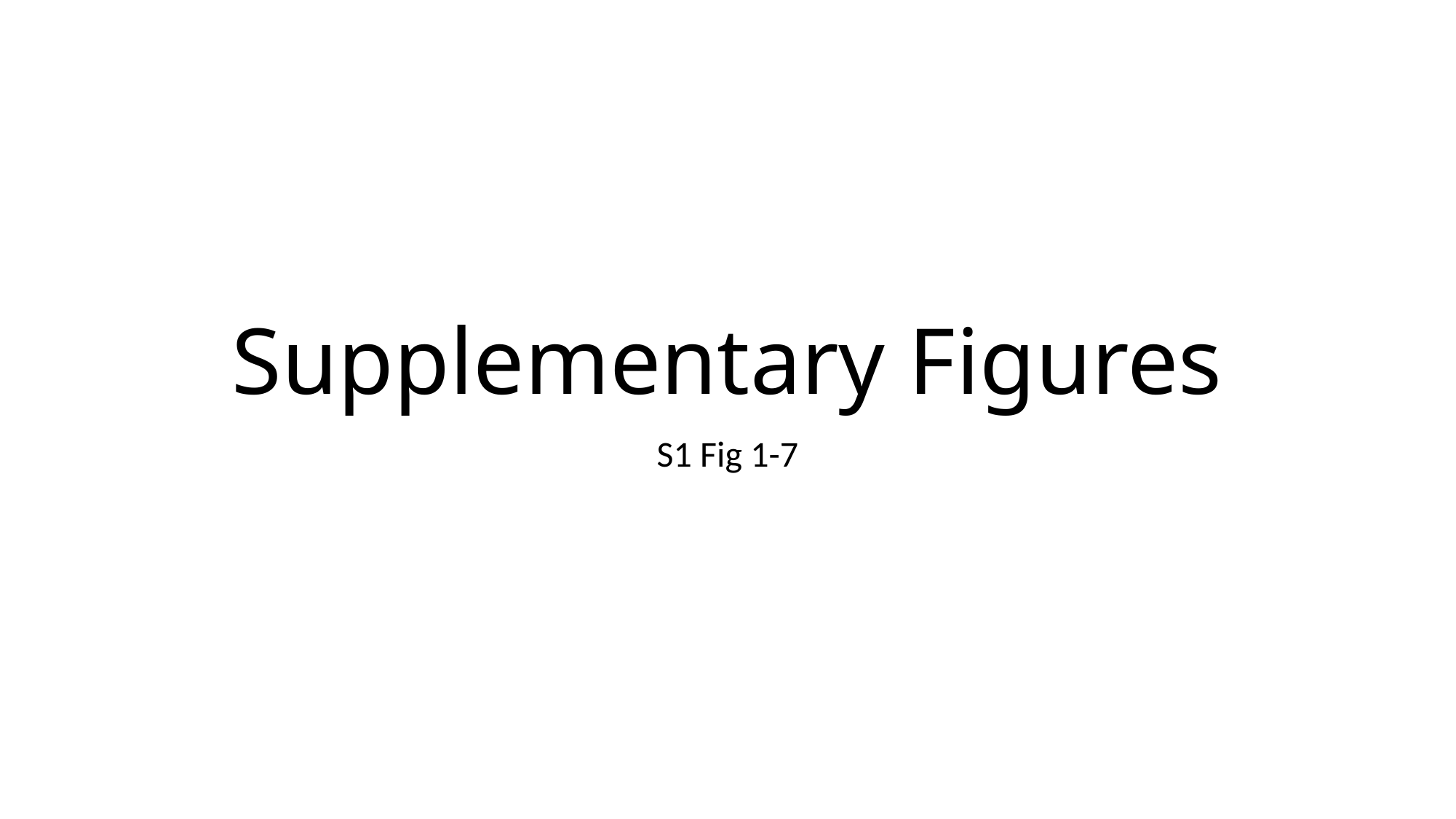

# Supplementary Figures
S1 Fig 1-7

## Slide 2
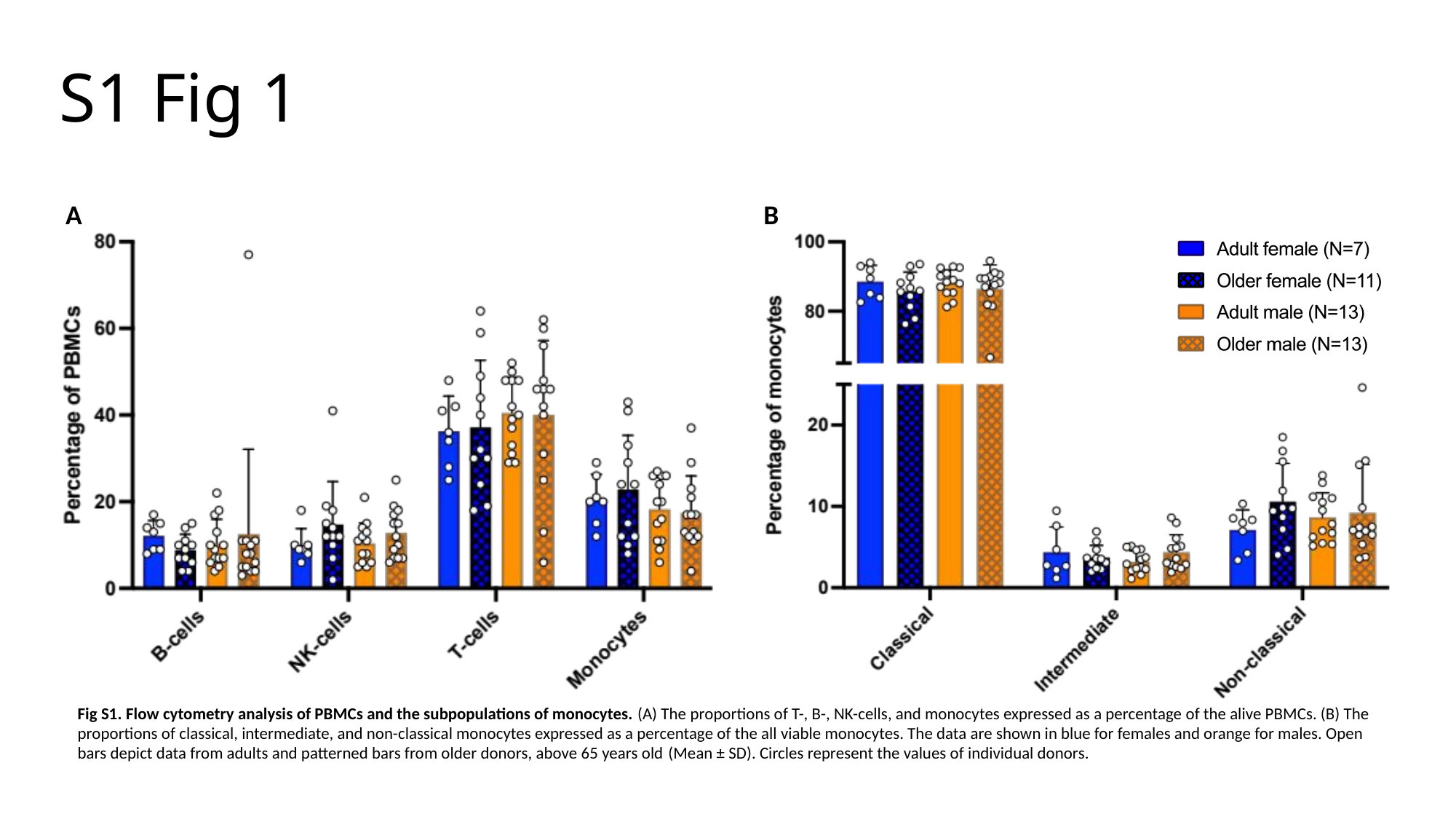

# S1 Fig 1
A						 B
Fig S1. Flow cytometry analysis of PBMCs and the subpopulations of monocytes. (A) The proportions of T-, B-, NK-cells, and monocytes expressed as a percentage of the alive PBMCs. (B) The proportions of classical, intermediate, and non-classical monocytes expressed as a percentage of the all viable monocytes. The data are shown in blue for females and orange for males. Open bars depict data from adults and patterned bars from older donors, above 65 years old (Mean ± SD). Circles represent the values of individual donors.

## Slide 3
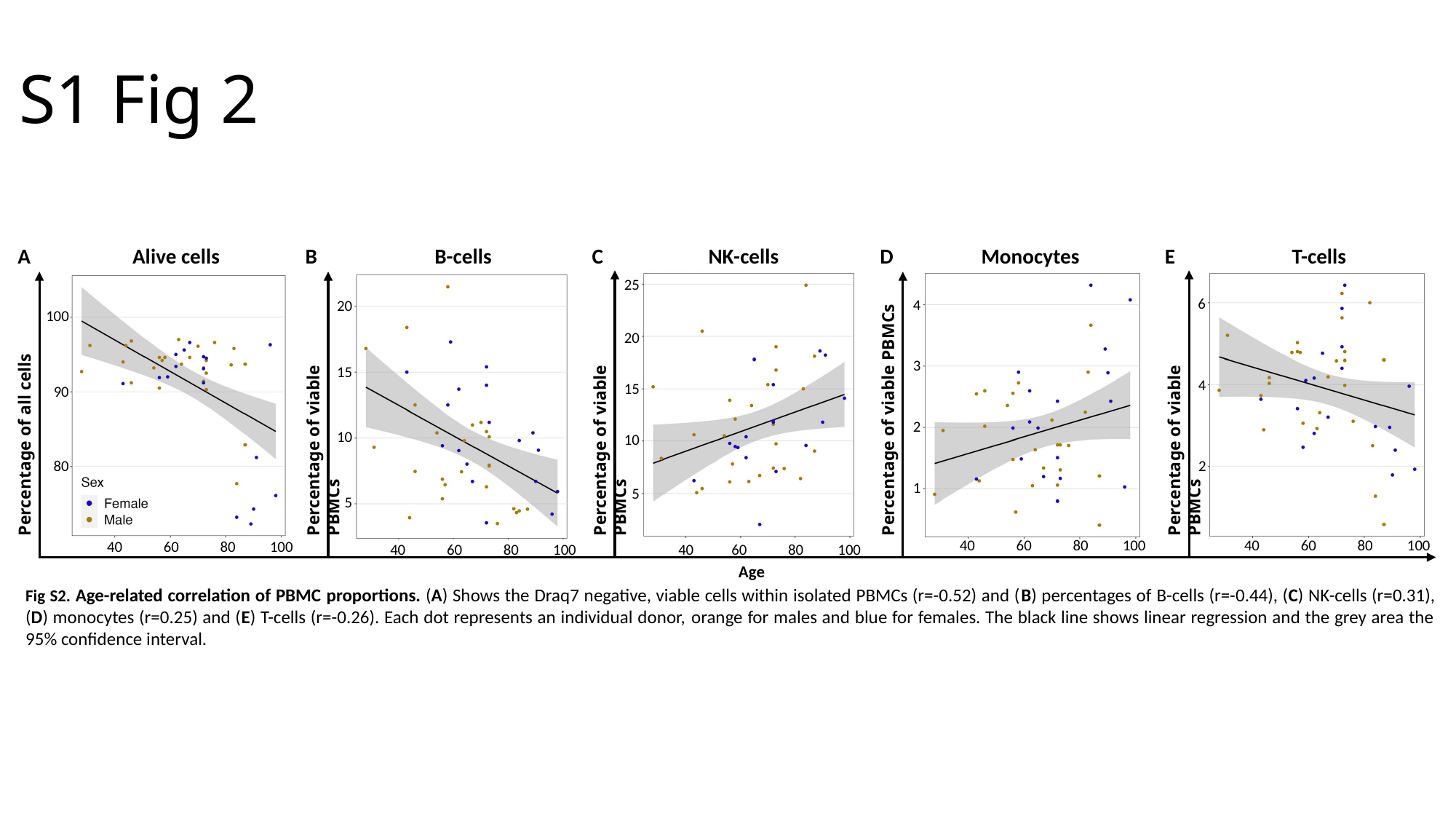

# S1 Fig 2
A
Alive cells
B
B-cells
C
NK-cells
D
Monocytes
E
T-cells
25
6
4
20
100
20
3
15
4
15
90
Percentage of viable PBMCs
Percentage of all cells
Percentage of viable PBMCs
Percentage of viable PBMCs
Percentage of viable PBMCs
2
10
10
80
2
1
5
5
40 60 80 100
40 60 80 100
40 60 80 100
40 60 80 100
 40 60 80 100
Age
Fig S2. Age-related correlation of PBMC proportions. (A) Shows the Draq7 negative, viable cells within isolated PBMCs (r=-0.52) and (B) percentages of B-cells (r=-0.44), (C) NK-cells (r=0.31), (D) monocytes (r=0.25) and (E) T-cells (r=-0.26). Each dot represents an individual donor, orange for males and blue for females. The black line shows linear regression and the grey area the 95% confidence interval.

## Slide 4
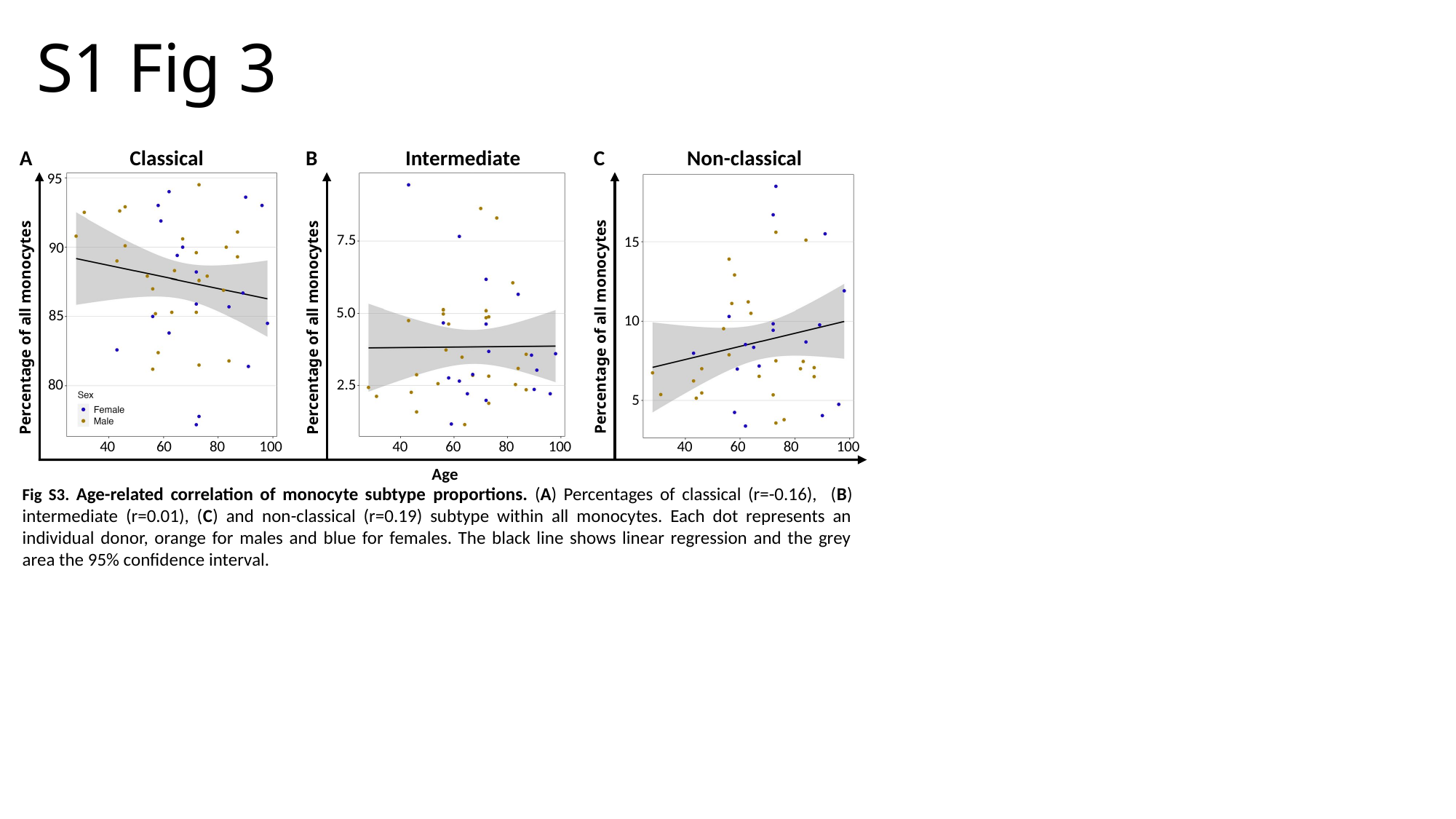

# S1 Fig 3
A
Classical
B
Intermediate
C
Non-classical
95
7.5
15
90
Percentage of all monocytes
5.0
Percentage of all monocytes
Percentage of all monocytes
85
10
80
2.5
5
40 60 80 100
40 60 80 100
40 60 80 100
Age
Fig S3. Age-related correlation of monocyte subtype proportions. (A) Percentages of classical (r=-0.16), (B) intermediate (r=0.01), (C) and non-classical (r=0.19) subtype within all monocytes. Each dot represents an individual donor, orange for males and blue for females. The black line shows linear regression and the grey area the 95% confidence interval.

## Slide 5
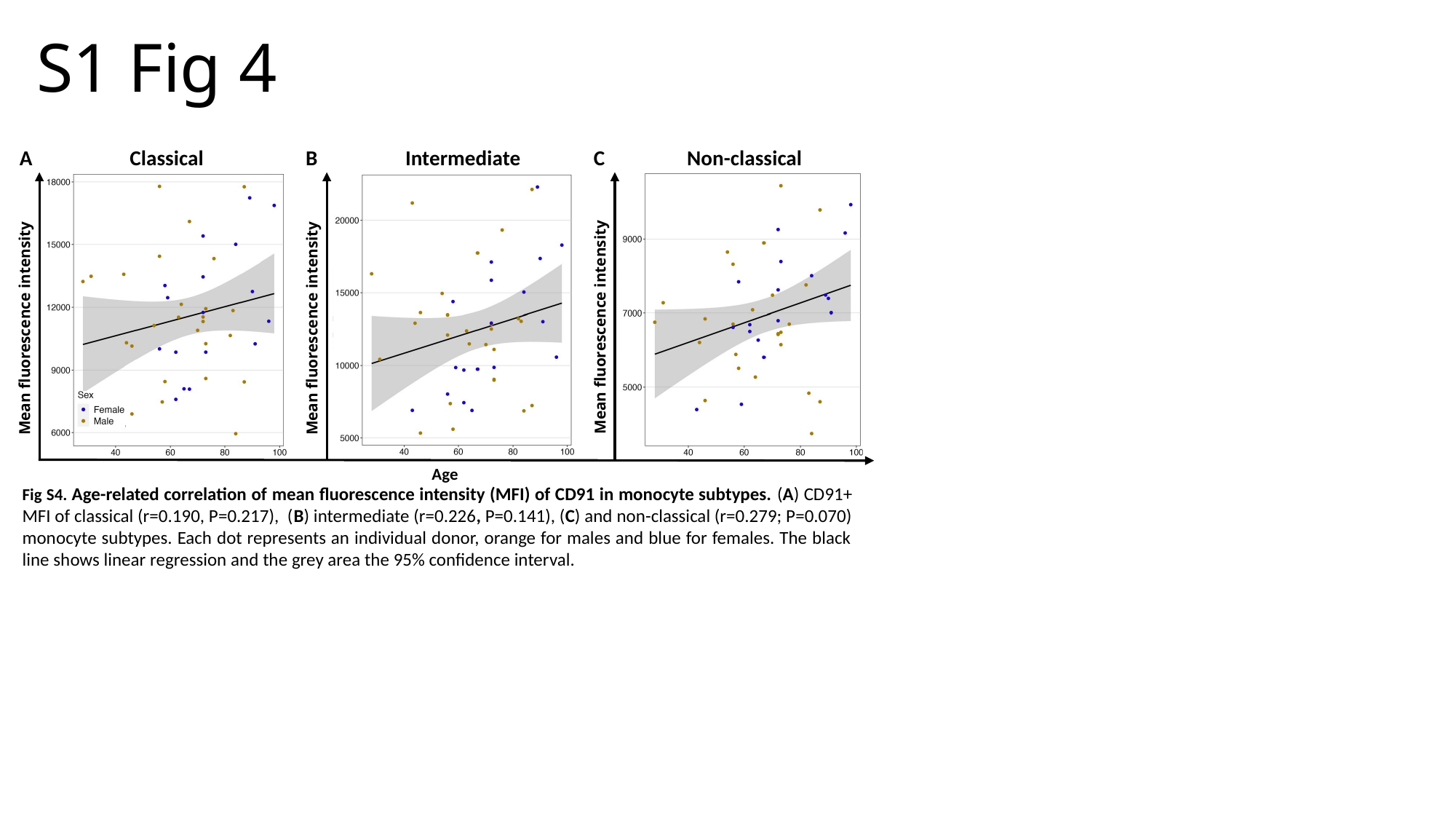

# S1 Fig 4
A
Classical
B
Intermediate
C
Non-classical
Mean fluorescence intensity
Mean fluorescence intensity
Mean fluorescence intensity
Age
Fig S4. Age-related correlation of mean fluorescence intensity (MFI) of CD91 in monocyte subtypes. (A) CD91+ MFI of classical (r=0.190, P=0.217), (B) intermediate (r=0.226, P=0.141), (C) and non-classical (r=0.279; P=0.070) monocyte subtypes. Each dot represents an individual donor, orange for males and blue for females. The black line shows linear regression and the grey area the 95% confidence interval.

## Slide 6
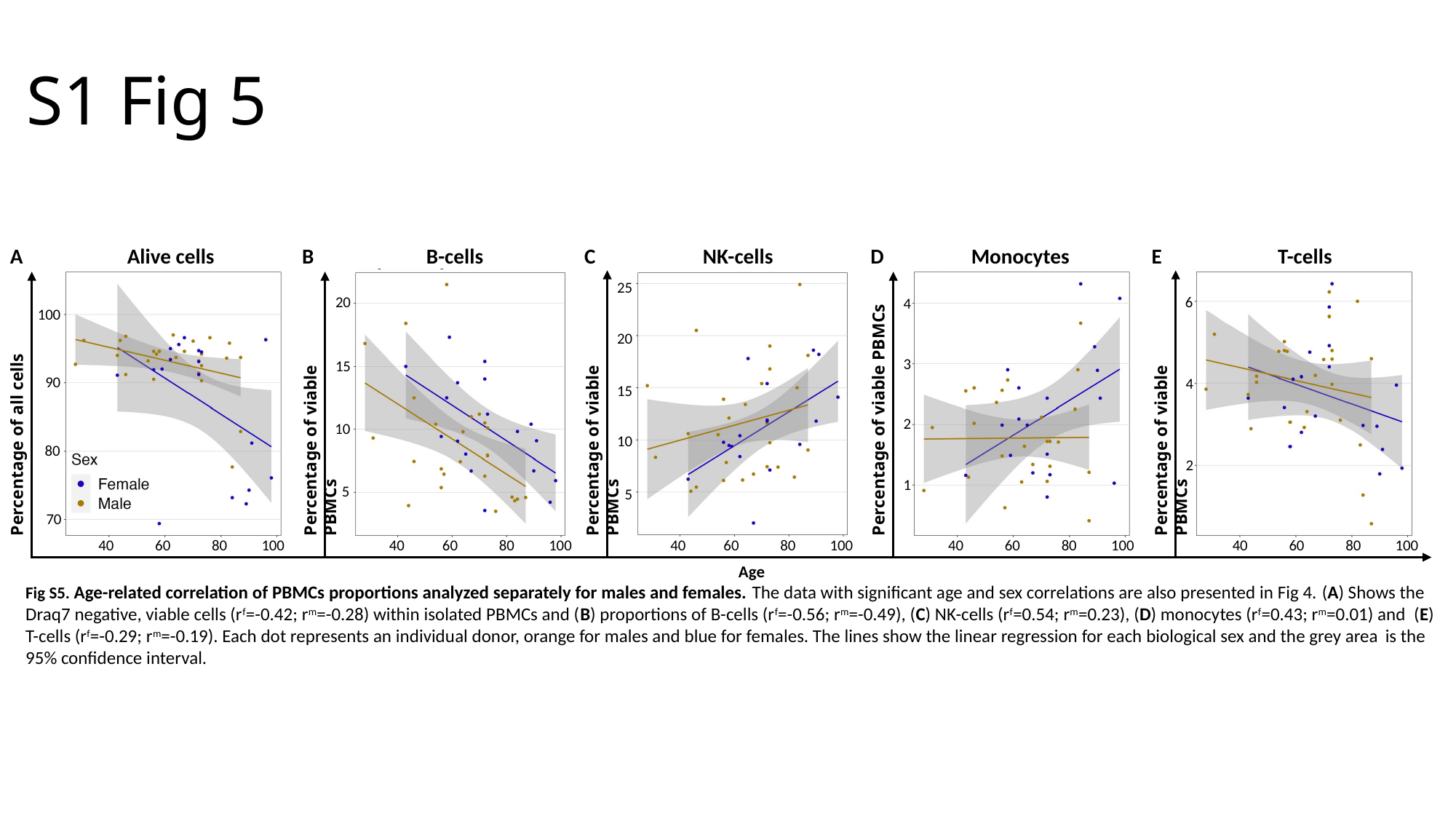

# S1 Fig 5
A
Alive cells
B
B-cells
C
NK-cells
D
Monocytes
E
T-cells
25
6
20
4
100
20
3
15
90
4
15
Percentage of viable PBMCs
Percentage of all cells
Percentage of viable PBMCs
Percentage of viable PBMCs
Percentage of viable PBMCs
2
10
10
80
2
1
5
5
70
40 60 80 100
40 60 80 100
40 60 80 100
40 60 80 100
40 60 80 100
Age
Fig S5. Age-related correlation of PBMCs proportions analyzed separately for males and females. The data with significant age and sex correlations are also presented in Fig 4. (A) Shows the Draq7 negative, viable cells (rf=-0.42; rm=-0.28) within isolated PBMCs and (B) proportions of B-cells (rf=-0.56; rm=-0.49), (C) NK-cells (rf=0.54; rm=0.23), (D) monocytes (rf=0.43; rm=0.01) and (E) T-cells (rf=-0.29; rm=-0.19). Each dot represents an individual donor, orange for males and blue for females. The lines show the linear regression for each biological sex and the grey area is the 95% confidence interval.

## Slide 7
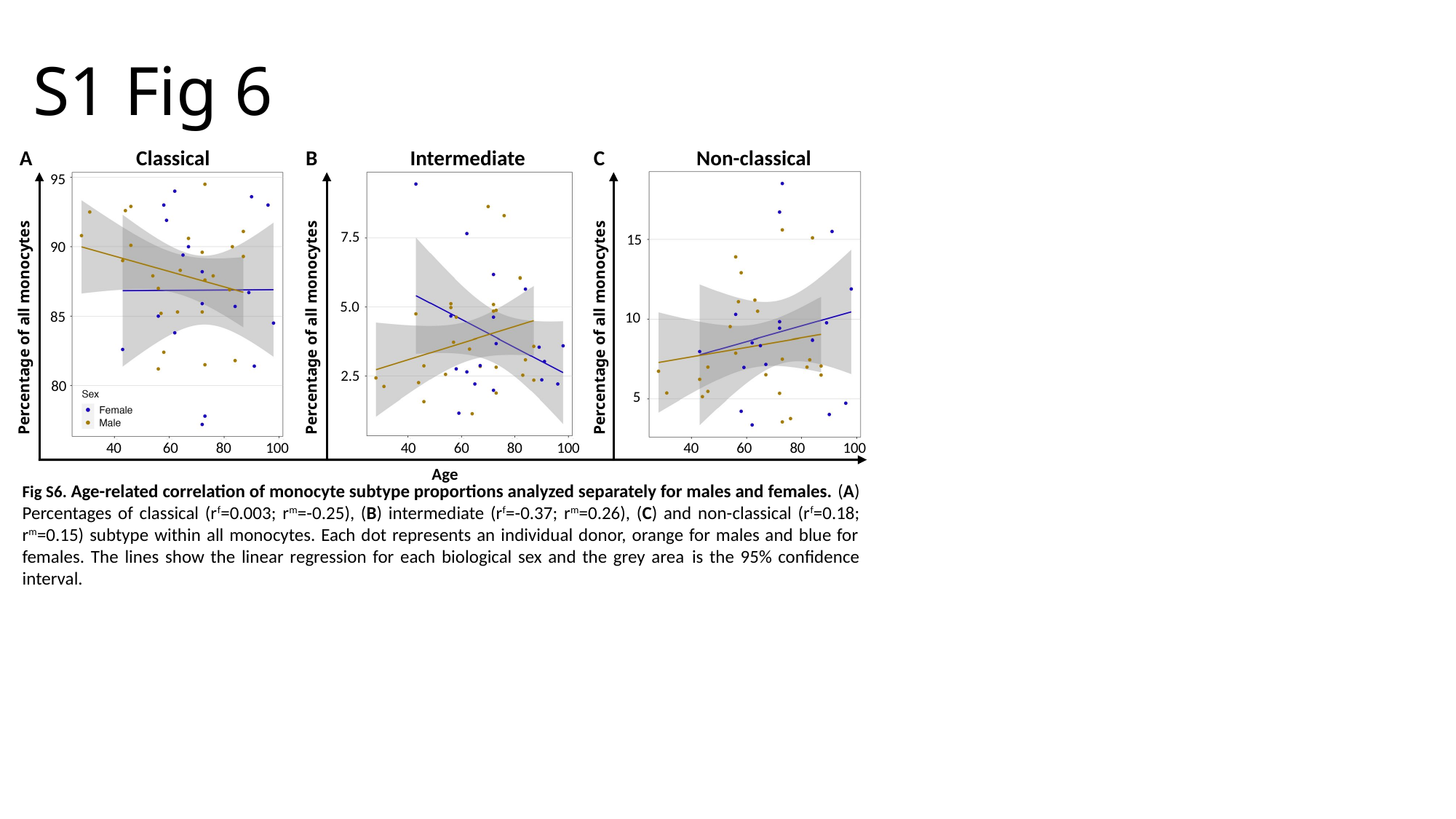

# S1 Fig 6
A
Classical
B
Intermediate
C
Non-classical
95
7.5
15
90
Percentage of all monocytes
5.0
Percentage of all monocytes
Percentage of all monocytes
85
10
2.5
80
5
40 60 80 100
40 60 80 100
40 60 80 100
Age
Fig S6. Age-related correlation of monocyte subtype proportions analyzed separately for males and females. (A) Percentages of classical (rf=0.003; rm=-0.25), (B) intermediate (rf=-0.37; rm=0.26), (C) and non-classical (rf=0.18; rm=0.15) subtype within all monocytes. Each dot represents an individual donor, orange for males and blue for females. The lines show the linear regression for each biological sex and the grey area is the 95% confidence interval.

## Slide 8
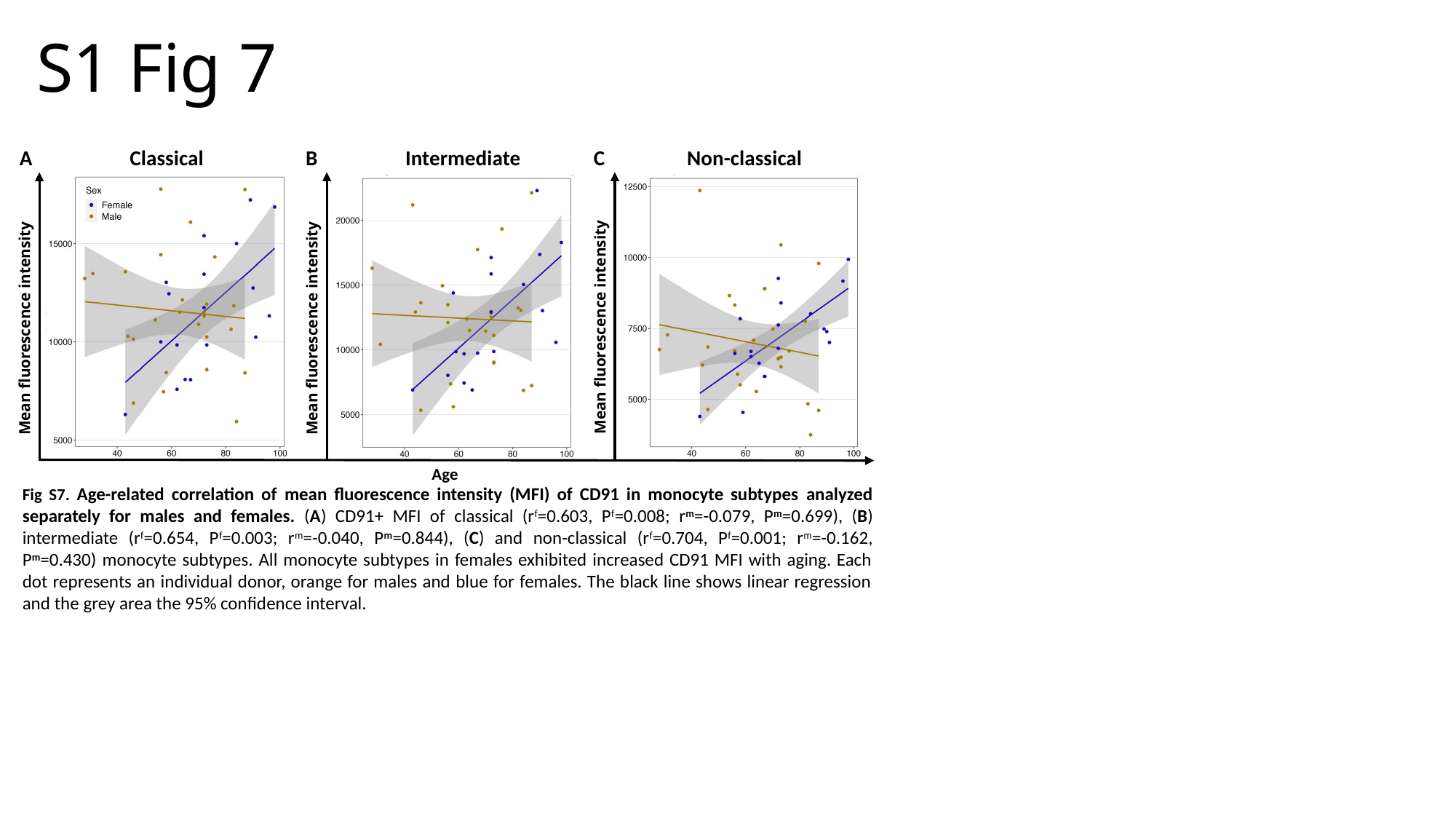

# S1 Fig 7
A
Classical
B
Intermediate
C
Non-classical
Mean fluorescence intensity
Mean fluorescence intensity
Mean fluorescence intensity
Age
Fig S7. Age-related correlation of mean fluorescence intensity (MFI) of CD91 in monocyte subtypes analyzed separately for males and females. (A) CD91+ MFI of classical (rf=0.603, Pf=0.008; rm=-0.079, Pm=0.699), (B) intermediate (rf=0.654, Pf=0.003; rm=-0.040, Pm=0.844), (C) and non-classical (rf=0.704, Pf=0.001; rm=-0.162, Pm=0.430) monocyte subtypes. All monocyte subtypes in females exhibited increased CD91 MFI with aging. Each dot represents an individual donor, orange for males and blue for females. The black line shows linear regression and the grey area the 95% confidence interval.
